# Metabolic Expenditures of Lunge Feeding Rorquals Across Scale: Implications for the Evolution of Filter Feeding and the Limits to Maximum Body Size

**DOI:** 10.1371/journal.pone.0044854

**Published:** 2012-09-14

**Authors:** Jean Potvin, Jeremy A. Goldbogen, Robert E. Shadwick

**Affiliations:** 1 Department of Physics, Saint Louis University, Saint Louis, Missouri, United States of America; 2 Cascadia Research Collective, Olympia, Washington, United States of America; 3 Department of Zoology, University of British Columbia, Vancouver, British Columbia, Canada; Texas A&M University-Corpus Christi, United States of America

## Abstract

Bulk-filter feeding is an energetically efficient strategy for resource acquisition and assimilation, and facilitates the maintenance of extreme body size as exemplified by baleen whales (Mysticeti) and multiple lineages of bony and cartilaginous fishes. Among mysticetes, rorqual whales (Balaenopteridae) exhibit an intermittent ram filter feeding mode, lunge feeding, which requires the abandonment of body-streamlining in favor of a high-drag, mouth-open configuration aimed at engulfing a very large amount of prey-laden water. Particularly while lunge feeding on krill (the most widespread prey preference among rorquals), the effort required during engulfment involve short bouts of high-intensity muscle activity that demand high metabolic output. We used computational modeling together with morphological and kinematic data on humpback (*Megaptera noveaangliae*), fin (*Balaenoptera physalus*), blue (*Balaenoptera musculus*) and minke (*Balaenoptera acutorostrata*) whales to estimate engulfment power output in comparison with standard metrics of metabolic rate. The simulations reveal that engulfment metabolism increases across the full body size of the larger rorqual species to nearly 50 times the basal metabolic rate of terrestrial mammals of the same body mass. Moreover, they suggest that the metabolism of the largest body sizes runs with significant oxygen deficits during mouth opening, namely, 20% over maximum 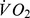
at the size of the largest blue whales, thus requiring significant contributions from anaerobic catabolism during a lunge and significant recovery after a lunge. Our analyses show that engulfment metabolism is also significantly lower for smaller adults, typically one-tenth to one-half 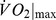. These results not only point to a physiological limit on maximum body size in this lineage, but also have major implications for the ontogeny of extant rorquals as well as the evolutionary pathways used by ancestral toothed whales to transition from hunting individual prey items to filter feeding on prey aggregations.

## Introduction

The extreme body size of baleen whales (Mysticeti) is generally attributed to the overall energetic efficiency of bulk filter feeding [1]. Instead of hunting single prey items which is typified by the raptorial and suction feeding strategies in toothed whales (Odontoceti), baleen whales are obligate suspension filter feeders that engulf large quantities of prey-laden water. By processing vast amounts of small prey in bulk, baleen whales are thought to reap the rewards of an abundant resource using a more efficient feeding mechanism [2], [3]. However, the energetics of foraging in cetaceans has yet to be fully assessed, especially with respect to microphagy in large mysticetes. Considering that some baleen whale species represent the largest animals to have ever lived, exploring the metabolic expenditures of feeding in baleen whales may shed light on the evolution of gigantism as well as the potential energetic limits to body size.

Baleen whales exhibit a variety of filter feeding mechanisms, with distinct modes occurring in different mysticete lineages [2], [4]. Bowhead and right whales (Balaenidae) are continuous ram feeders that exploit patches of copepods at slow and steady speeds [5], using massive tongues to direct incoming prey-laden water along parallel racks of baleen [6], [7]. Gray whales (Eschrichtiidae), on the other hand, possess relatively smaller, but more mobile tongues that can be depressed to suction-feed on benthic invertebrates [4]. In contrast to both balaenids and eschrichtiids, rorqual whales (Balaenopteridae) exhibit highly expendable ventral pouches along with extensible tongues that invert into capacious sacs, to enable the engulfment of a large and discrete volume of prey and water [8], [9]. After the jaws have closed around the engulfed water, prey is filtered using plates of baleen as water is purged from the now inflated ventral pouch. Although different rorqual species exhibit a wide diversity in prey preferences (fish, copepods, squid, crabs, etc.), foraging strategy [10]–[12], and ecological niche [13], [14], krill represents one of the most common prey types [15]. In general, engulfment is preceded by prey-approach, and then followed by an obligatory filter phase which may also allow some degrees of metabolic recovery. Depending on the depth and quality of the krill patch, several lunge feeding sequences of pre-approach, engulfment and filtering/recovery may be repeated during a single dive.

Lunge feeding requires a coordinated suite of anatomical and biomechanical adaptations [9], [16]. In addition to the foldable (and muscularized) ventral pouch [17]–[19], the rorqual engulfment apparatus is comprised of flexible jaw joints that enable the rotation of the mandibles, which directly increase the area of the mouth [20], [21] and therefore the flux of water into the oropharyngeal cavity [22]. The engulfment capacity of the ventral pouch is ultimately limited by the mechanical properties of the ventral groove blubber (VGB), a specialized blubber layer that is reversibly extensible up to several times its resting length [19]. Both the hard and soft tissue structures of the engulfment apparatus exhibit positive allometry, whereby the dimensions of these elements are relatively longer in larger animals [23]. As a consequence, mass-specific engulfment capacity increases with body size both within and among rorqual species [24]. Because larger rorquals have the ability to engulf relatively larger volumes of prey-laden water, overall feeding efficiency is significantly increased in larger whales [25].

Hydrodynamic modeling of engulfment parameterized with kinematic and morphological data indicates that lunge feeding comes at high energetic costs which are largely incurred from the engulfment and entrainment of a very large amount of water [25]–[27]. Such high energetic expenditures are significant enough to greatly reduce, in comparison with non lunge-feeding whales of similar size, the diving time in which foraging occurs [28]. This phenomenon has previously been addressed in the context of the *Theoretical Aerobic Dive Limit* (TADL), which is largely a function of the ratio of the metabolic expenditures during a dive to the oxygen storage capacity in blood, muscle and lungs [24], [28], [29]. What TADL doesn’t explain, however, is the fact that even though all large rorquals have similar maximal foraging diving times, namely, anywhere from 9 to 11 min. in humpback (*Megaptera noveaangliae*), fin (*Balaenoptera physalus*) and blue (*Balaenoptera musculus*) whales [24], their capacity to execute many lunges during a single dive declines significantly with body size, i.e., from 12, to 7, to 4 lunges per dive in those same three species respectively [24]. Although there appears to be enough oxygen storage capacity to meet the energetic demands of foraging at all body sizes and to the same depths, there seems to be a more proximate limit associated with the lunge component of foraging at the largest sizes, in particular with the *rate* of energy expenditure during the engulfment stage. Here we explore the hypothesis that reduced lunge frequency at the larger sizes is, at least for these three large Rorqual species, a reflection of the metabolic power requirements of engulfment becoming so high to involve significant oxygen deficits and muscle fatigue during each lunge. Such increased power requirements follow from the allometry of the skull which permits the engulfment of ever greater masses of water and prey (relative to body mass [23]) and at greater energetic costs, but as performed over nearly unchanging engulfment durations constrained by the escape time scales of the prey [27]. Reduced diving capacity and lunge frequency during foraging has major consequences for rorqual ecology and evolution because it begins to decrease the prime benefit of bulk feeding, namely a high energetic efficiency, by limiting access duration to high quality prey resources at depth [25].

Understanding the relative importance of total energetic expenditures versus power output (the rate of expenditure) requires the consideration of the various time scales characterizing the stages of a lunge. Within the context of a single foraging dive where durations and expenditures for diving, prey-approach, engulfment and filtering/recovery are all accounted for, the (averaged) metabolic rates and corresponding energetic expenditures are found to be only slightly higher than those of steady swimming [25]. When extrapolated over several months of intense foraging during the summer, these power requirements of foraging are still low compared to the rate of energy intake such that it facilitates the deposition of substantial lipid stores needed for long distance migration and reproduction [30]. However, and at the smaller time scale of the engulfment stage (of about 10 seconds or less), there is a requirement for short bouts of intense muscle activity and for a metabolic output during which a large body of water (both external and internal) needs to be quickly set into motion, a process for which muscle fatigue and consequent recovery may become a limitation. Although previous research has focused on estimating the energetic cost of lunge feeding [24], [25], [27], scant attention has been paid to the power output required by this extreme feeding strategy. As a result, both its ecological and evolutionary implications remain poorly understood. Here we address this dearth in our knowledge of rorqual foraging energetics with a new, high time-resolved hydro-mechanical model based on previous work [26], [27], but now capable of accurately distinguishing the changing physics of the various sub-stages of engulfment.

The obvious impossibility of studying the energetics of large whales in a laboratory setting makes computer modeling the only tool available for assessing the relevant factors driving the metabolic requirements of engulfment. These include the work done by the locomotor muscles for swimming and by the musculature embedded in the VGB for accelerating the engulfed water mass. An additional factor is the large amount of energy a whale typically loses to hydrodynamic drag. Given the substantial speeds imparted to the engulfed mass, and the need for coordinating the dynamics of a lunge in order to optimize engulfment volume [26], engulfment modeling must also include a hydrodynamic model that is coupled to the forces generated by VGB musculature. Here we quantify the effects of these factors over the adult sizes of humpback, fin and blue whales, as well as at one size of the much smaller minke whale (*Balaenoptera acutorostrata*), all investigated at a level of detail that we believe yields the correct physics across all body sizes and over the time scales of prey escape and engulfment [27]. This model now accounts for the water being engulfed anterior to the temporomandibular joint (TMJ) in between the mandibles, in addition to the water engulfed posterior to the TMJ [26], as well as the effects arising from the wake re-contacting a decelerating whale during mouth closure. The new model thus removes some of the uncertainties that required input sensitivity analyses in previous studies [24], [25] and which inevitably reduced the prediction ability of the model.

The simulated engulfment metabolic rates (EMR) are compared with various standardized measures of metabolic effort, including the *Basal Metabolic Rate* (BMR) and the *Rorqual Average Active Metabolic Rate* (RAAMR), a new assessment of active metabolism specific to balaenopterids. New estimates of the metabolic expenditures sustained during the prey-approach (PAMR) and the filter/recovery stages (F/RMR) will be discussed as well. Our analyses show that the metabolic expenditures of engulfment (averaged over mouth-opening time scales) significantly change with body size, from about equal to RAAMR at 10 m (a medium-sized humpback whale or small fin whale), to about 3.7 RAAMR at 27 m (a very large blue whale). Generally, the expenditures sustained prior to, and following engulfment (i.e., PAMR and L/RMR) are similar to RAAMR within 50%. Comparisons of maximum *instant* EMR (rather than time-averaged EMR) show an even steeper power requirement: namely, and when compared with the BMR of terrestrial mammals of the same mass (BMR(terr)), an increase from about 8 BMR(terr) at 8 m body lengths, to 48 BMR(terr) at 27 m. When further compared in terms of maximum aerobic capacity
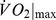
(as represented by the *Maximum Metabolic Rate* (MMR)), the magnitude of maximum instantaneous EMR suggests substantial oxygen deficits at the largest body sizes (27 m), with expenditure rates exceeding 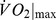
by 20% during a good portion the mouth opening stage. Characterized as a *supramaximal* type of effort [31], we suggest that such expenditure levels can only be met by significant contributions of anaerobic metabolism for which muscular fatigue is more important and metabolic recovery longer (i.e., recovery after each lunge during filtering *and* after each dive). Interestingly, a simulated 33 m blue whale shows required power outputs as high as 80% above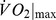, and maintained over durations that are long enough to explain why it is perhaps non-extant. On the other hand, engulfment metabolism requires significantly lower outputs, namely 0.1 to 0.5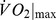, at the small body sizes of all three large species and at the one simulated size of the minke whale, the smallest of the Rorquals (with body lengths of less than 10 m). As often characterized as *light submaximal* and *heavy submaximal* [31], such effort levels can be sustained for longer periods of activity and over several lunges during a single dive. These drastically reduced metabolic demands at smaller body sizes are similar to those of non-feeding swimming, and bring about interesting questions about the ontogeny of extant rorquals, as well as the evolution and physiological limits to different lunge feeding modes.

## Materials and Methods

Engulfment metabolic rate calculations are based on a simulation of the forces at play during mouth opening and closure. Determination of the metabolic rates follows after tallying the corresponding (mechanical) energy and power while taking into account assumed metabolic efficiencies. The hydrodynamic model used for engulfment simulations, the *Basic Lunge Feeding model* (or BLF for short; version 3.0), is a significant upgrade of a model originally devised a few years ago [26], [27]. Although the BLF dynamically couples both structure (whale) and fluid (flow), its one-dimensional fluid dynamics and simple (bulk) VGB force modeling makes it far less complex than computationally detailed models where changing body shape morphometrics is digitized into structure meshes about which three-dimensional fluid simulations are performed [32], or for which structural (elastic) strains are computed with finite element methods [33]. Simplicity is allowed here by the physics of lunge feeding, where most of the relevant fluid masses being displaced are moving in only one direction, i.e., forward, along the whale’s trajectory. Moreover, the fluid-pushing forces supplied by the whale’s VGB are active pulls by muscles of (quantitatively) unknown distributions and densities, rather than elastic-based strains of a well-defined rigid structure (the skeleton).

The general principles, upgrades and approximations of the BLF model will be summarized in the following paragraphs and its mathematics discussed in *Modeling Details* (which follows the *Conclusion*). Estimates of the metabolic output sustained during prey-approach, as well as that of the active metabolic rate specific to Rorquals, will also be discussed below while the filter/recovery metabolic rates will be described in the *Discussion*. A list of the symbols and acronyms can be found in Text S1.

### Hydrodynamic Model

#### Input parameters and scope

**Figure 1:**
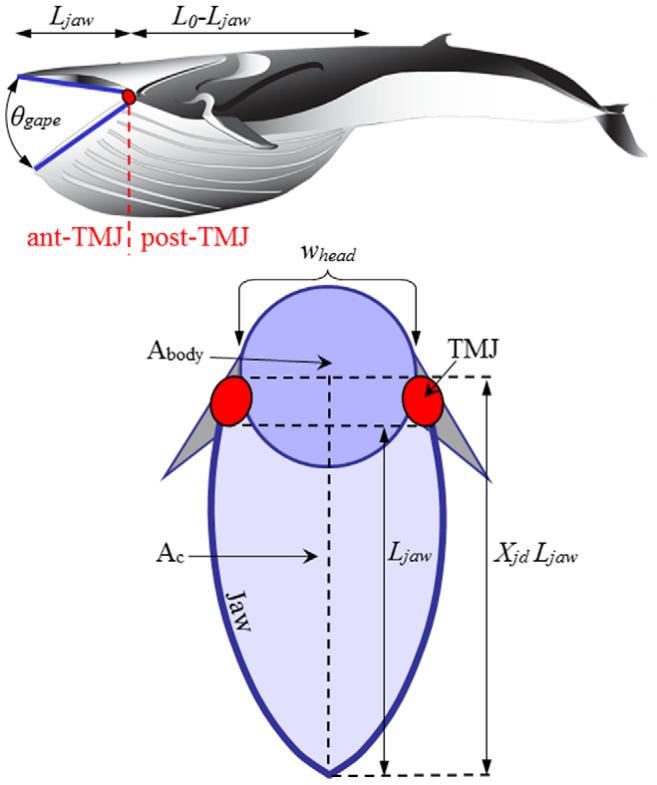
Dimensional characteristics of the mouth apparatus and ventral cavity.

The BLF is informed by a variety of inputs, including six entries based on the known morphology of the VGB and skull (Figure 1 and Tables 1, 2, 3, 4). On the other hand, one table input, as well as the validation of the speeds calculated at each species’ average body size, have relied on the velocity data collected by tags deployed on humpback, fin and blue whales lunging at depth along horizontal or inclined tracks (both “uphill” or “downhill” tracks; see discussions in [11], [25], [34], [35]). Finally, the overall sequence of body shapes being simulated [26], [27] is similar to what is shown in the film and photographic record of rorquals feeding on krill at or near the surface. Although these data provide many useful insights, they obviously limit the applicability of the model to lunges towards slow-moving prey along horizontal or straight but inclined trajectories. As observed with many rorqual species, lunge-feeding is a plastic behavior where diverse combinations of lunge speeds, body-rolling maneuvers, motion headings and gape angle dynamics are used to exploit different prey types and distributions [10], [11], including the bubble-netting technique used by humpback whales to lunge-feed vertically towards fish [12]. However, the types of lunges discussed here represent the most common foraging strategy among rorquals and consequently their simulations should provide a realistic assessment of engulfment energetics.

**Table 1:** BLF3 simulation inputs – blue whales.

L­_body_ (m)†	L_0_ (m)†	L_jaw_ (m)†	w_head_ (m)†	M_c_ (kg)†	A_body_ (m^2^)†	V_c_(0) (m/s)†	F_thrust_ (N)	k_open_
19	10.5	3.37	2.11	36 348	4.29	2.77	3559	14.4
22.1	12.58	4.17	2.53	61 318	6.41	3.23	4750	13.5
25.2 (average length)	14.71	5.02	2.96	96 568	9.09	3.68	6103	12.9
27	15.98	5.54	3.22	122 605	10.92	3.94	6963	12.6
33 (non-extant)	20.30	7.34	4.09	245 499	18.63	4.82	10 215	11.9

† References 24, 39, 55–62.

Inputs applying to all body lengths: ρw = 1025 kg/m3, dt = 0.01 s, θgapemax = 78°, kam = 0.2, kopen/kclose = 1.83, CDopen = 0.3, CDclose = 0.5, CDbody = 0.05, Xjd = 1.00, Γ = 1.2, Vw(0) = 0 m/s, χ = 1.0, φ = 1.6 (0≤ t ≤0.66 topen), φ = 0.0 (0.66 topen < t ≤ tengulf). hsync is computed from Fsync/Xjd = (θgapemax/Γ) sin θgapemax Ljaw/(L0 - Ljaw) (Potvin et al [27]).

L−body = Body length; L0 = Length of the VGB; Ljaw = Length of the palate; whead = Width of the skull; Mc = Body mass; Abody = Mean cross section area of the (empty) body; Vc(0) = Whale speed at beginning of mouth opening; Fthrust = Fluking thrust during mouth opening (equation 20); kopen = Reaction constant (mouth opening). The symbols are further explained in the text or in Text S1.

#### Synchronized engulfment

The model uses the (coupled) Newtonian motion equations of the whale body and engulfed water, as constrained by an engulfment scenario in which, and as suggested by the film record, the filling of the cavity sections posterior and anterior to the TMJ are sequential rather than simultaneous (see Diagram 1 in [27]). In what is described here as *Synchronized Engulfment* (SE), a whale is assumed to first fill its cavity post-TMJ until maximum gape. This is followed, during mouth closure, by the filling of the buccal cavity anterior to the TMJ, as well as by the engulfed mass moving at the speed of the whale - a state of motion herein termed as *equivelocity*. *Equivelocity* is an important simulation constraint as it implies the absence of flows out of the cavity past the moment of maximum gape, as shown by the film record of surface lunges. On the other hand, the term “synchronized” in SE refers to the coordinated use of swimming muscles and engulfment apparatus (VGB muscles, temporalis, sternomandibularis) being individually modulated to ensure sequential cavity filling [27], possibly according to the inputs of a recently identified sensory organ located at the mandibular symphysis [16].

Computer simulations suggest that the expansion of the ventral pouch is actively resisted by the eccentric contraction of the VGB musculature [26] rather than passively by VGB elasticity [19]. The engulfed water is thus accelerated forward from inside the open mouth and therefore represents an additional source of drag (engulfment drag) to that generated by the flow deflected around the body (shape drag). This concept of active (captured) flow control is supported by photographic evidence showing the lack of sufficient VGB distension at mouth closure [36], suggesting that the VGB is not stretching enough to enter the high stiffness region of the stress-strain curve obtained during *in vitro* tests on fin whale VGB [19]. We note, however, that VGB elasticity could play a minor role during and after engulfment as an energy absorber against the sloshing of engulfed water within the ventral pouch.

SE provides important constraints on body and fluid dynamics which simplifies the model without unduly compromising its accuracy. It provides also quantitative relationships among the model’s dynamical variables (such as engulfment duration), in terms of not only time and body dimensions but also of escape modes of the prey. These constraints and relationships are further discussed in [27] and summarized in *Modeling* **Details.**

### Forces at Play

**Figure 2:**
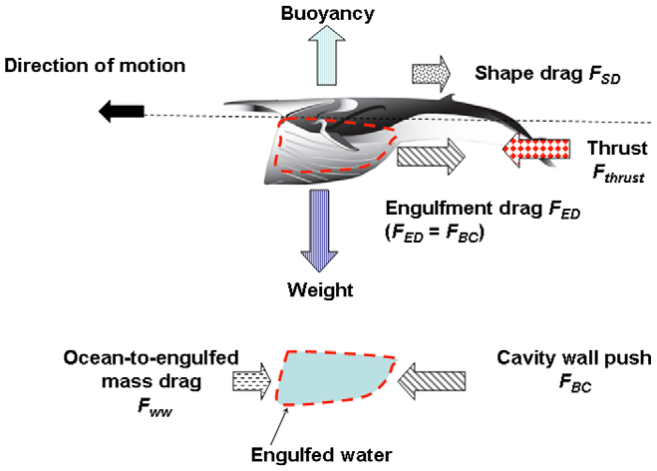
Forces acting on the whale body and engulfed mass.

The BLF model is a numerical scheme that computes the accelerations and speeds of the whale’s body (*a_c_* and *V_c_*) and its engulfed mass (*a_w_* and *V_w_*) as both interact with each other and with the surrounding fluid (Figure 2). The forces acting on the body consist in the following: the buccal cavity wall force (*F_BC_*), which by virtue of Newton’s third law of motion is equal in magnitude to the engulfment component of hydrodynamic drag (*F_ED_*); the so-called “shape” component of drag (*F_SD_*), arising from the flow deflection around the whale’s body; the tail thrust force (*F_thrust_*), and finally the weight-subtracted buoyancy force (*F_ext_*). The forces acting on the engulfed mass include the force *F_BC_* generated within the buccal cavity walls and mostly acting on its posterior-most section; and the “ocean-to-engulfed mass” drag *F_ww_*, acting on its ocean-facing end (Figure 2). The latter parameterize the effects of fluid pressure buildup under the palate, where the moving engulfed mass is meeting a static ocean. We note that using the force *F_BC_* acting in the bulk, rather than being unevenly distributed in magnitude and direction over the surface of the VGB, is a drastic oversimplification of reality, but one allowed by the overall one-dimensional (and incompressible) character of the moving engulfed mass (Figure 2).

**Figure 3:**
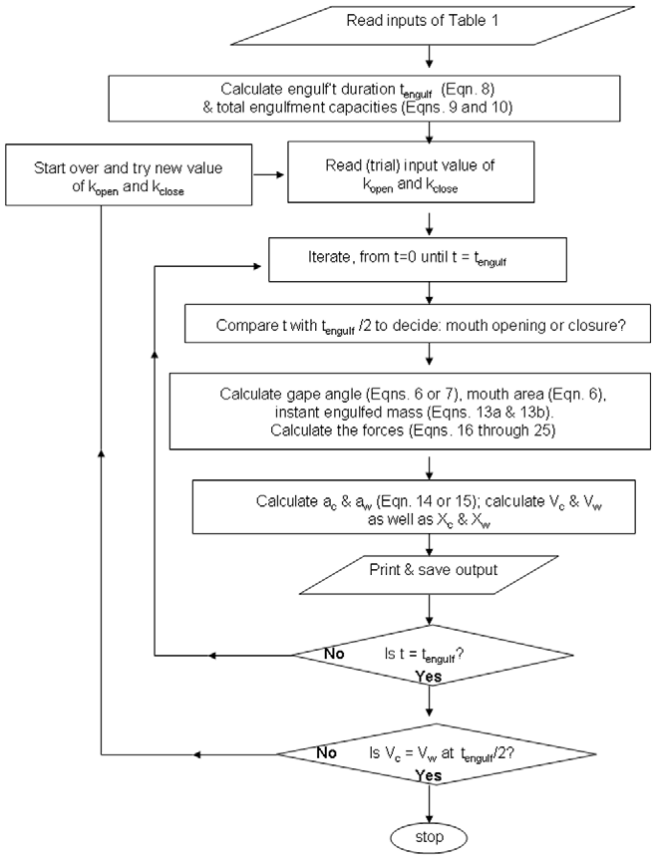
Logic flow diagram of the BLF model.

As shown in the flow chart of Figure 3, the BLF model simulates the full mouth-opening and closure sequence, beginning with the calculation (and prediction) of the duration of engulfment (*t_engulf_*; see equation 8 below) and total engulfed volumetric capacities (equations 9 and 10). This information is then fed into an iteration scheme aimed at calculating the forces, accelerations and speeds applied to the engulfed mass and body via Newton’s 2^nd^ law of motion, over each steps of a temporal sequence encompassing both mouth opening and closure. With the forces and speeds thus known, the mechanical work performed by each relevant force can be calculated at each time step and stored for the subsequent calculation of the metabolic rates.

### Modeling Metabolic Power Output

Metabolic power expenditures during engulfment are calculated from the BLF-simulated forces and mechanical energies related to muscle use for swimming thrust (*F_thrust_*) and buccal cavity push (*F_BC_*) (or engulfment drag (*F_ED_*)). Note that in Goldbogen et al. [24], the expenditures were based on the consideration of total drag and swimming thrust accordingly to a scenario of shape drag possessing an active component (see Appendix S1 of that paper). The expended energy for each force is obtained by summing the increments *ΔQ_T_^mech^ = F_thrust_ ΔX_c_* and *ΔQ_VGB_^mech^* = *F_BC_ ΔX_c_* over all steps of the simulation, with *ΔX_c_* corresponding to the distance travelled during each time step (*ΔX_c = _V_c_(t) dt*). The translation of mechanical energy into metabolic energy is achieved by dividing the sums *∑ΔQ_T_^mech^* and *∑ΔQ_VGB_^mech^* by efficiency factors, namely by 0.15 and 0.25, respectively [24]. The 0.15 efficiency factor applied to swimming thrust takes into account both losses from muscle activity and propulsive inefficiency, namely 75% and 10%-loss of the total metabolic power generated respectively [37], [38]; the 0.25 factor applied to *∑ΔQ_VGB_^mech^* accounts for the 75% metabolic losses incurred during VGB contractions. The rate of energy expenditure is calculated by dividing the work incurred by engulfment time (*t_engulf_*), namely, as *P_T_^metab^ = ∑ΔQ_T_^metab^/t_engulf_* and *P_VGB_^metab^ = ∑ΔQ_VGB_^metab^/t_engulf_*. The metabolic power outputs during the mouth opening and closure stages are calculated similarly, but integrated during their respective duration (i.e., over *t_engulf_/2*; see Equation 8 below), and the *instant* EMR (labeled EMR*) integrated over time intervals of 0.1 s.

The calculation of EMR also includes an estimate of the metabolic expenditures incurred by the rest of the body (i.e., besides swimming muscles and VGB musculature). This is achieved by adding to *P_T_^metab^ + P_VGB_^metab^* the metabolic output of the mass fraction *X* of those body parts that spend metabolic energy at rates obtained from the oft-used Active Metabolic Rate (AMR) [20], and the output of fraction *Y* that expend energy at rates similar to the Basal Metabolic Rate:
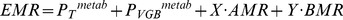
(1)


Previous studies that determined body composition in baleen whales estimates muscle mass at approximately 43% of total mass, in comparison with 25% blubber, 17% bone and 12% viscera [39]. Another study suggested locomotor musculature as representing 15% of total body mass [40]. If VGB musculature comprises about the same proportion as locomotor musculature (i.e., ∼15%), there remains about 43%–30% ∼ 13% of muscle mass which, along with viscera, could represent about 25% of body mass that may also function at higher metabolic rates. Given the presumed intensity of engulfment, and further assuming such tissues operating at rates similar to AMR, Equation 1 would thus use *X = 0.25* and *Y = 0*. Such values are obviously tentative but it turns out that they have minimal effects on the overall value of EMR.

We estimated AMR and BMR from allometric equations relating body mass to power, where *AMR = 3 BMR* [41], [42]. Following previous studies of marine mammal metabolism [1], [41], [43], BMR is assumed herein as twice that of terrestrial animals expressed via Kleiber’s scaling formula [44], [45]. Those two assumptions thus lead to:
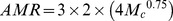
(2)


AMR-costs related to non-resting metabolic outputs by rorquals have been estimated either via assumed allometric formulations of AMR and BMR [42], [46], [47], or by assuming a specific form of the shape drag force [40]. These AMR-based studies have yielded estimates that differ by as much as 100%, depending on the specific AMR-BMR relationship and BMR-model being used, as discussed by Croll et al. [42]. The drag-based approach has yielded estimates lower than all AMR-based estimates, most likely due to an inadequate drag model that neglects the effects of surface-induced drag (i.e., ventilation and wave drag [48]), effects which are important in other marine mammals [49]. In the case of blue and fin whales, Equation 2 yields active metabolic rates that are lower than Lockyer [47] by about 50%, and higher than Croll et al. [42] by 50%. Further support for Equation 2 is presented below with another measure of active metabolic rate that is more specific to the bio-mechanics and ecology of rorquals.

### Rorqual Average Active Metabolic Rate (RAAMR) and Prey-Approach Metabolic Rate (PAMR)

Rorqual Average Active Metabolic Rate (RAAMR) is primarily based on a calculation by Bose and Lien [37] of the thrust generated by actual flukes using classical 2-dimensional airfoil theory with finite span corrections. Under an assumption of steady travel, power expenditures are calculated from the product of this calculated thrust (*F_thrust_^steady^*) and average speed of transport (*<V_c_>*), with the latter obtained here from long duration tracking data [50], [51]. In what follows, *<V_c_> = *2.16 m/s and 2.4 m/s for fin and blue whales respectively. Being extracted from data spanning days, these speeds reflect motions characteristic of not only long distance transit, but also of shorter events such as resting, mingling (socializing) and lunge-feeding. Although these activities are generally performed at different speeds, the long duration of the monitoring should be dominated mostly by the long-distance travel component of the track.

The calculations by Bose and Lien applied only to the fluke shape and size of a 14.5 m fin whale and, as such, needed to be extrapolated over body size and across species. This was done by first scaling the Bose-Lien result according to body drag, and thus to surface area (or, equivalently to body area *A_body_* (Figure 1 and Tables 1,2,3 4). As non-feeding swimming is often performed in groups of variously-sized individuals moving at the same speed, the transport speed *<V_c_>* is herein assumed as body size-independent. Moreover, non-feeding swimming speed tends to be relatively low across body size in many different taxa [52], [53]. The Bose-Lien results were also scaled across the body length of blue whales given their morphologic similarity with that of fin whales. This analysis was *not* extended to humpback whales given their significantly different fluke morphology.

A third extrapolation of the Bose-Lien thrust was performed with respect to travel speed, given the high flow speed these authors used (i.e., 4 to 12 m s^−1^) in comparison to the average travel speed measured by tracking. Considering the Bose-Lein data below 8 m s^−1^ and extrapolating down to 2 m s^−1^ yields the formula *F_thrust_^steady = ^1000 (1.216<V_c_> +0.0205<V_c_>^2^)*. The RAAMR that results is computed using a metabolic output model similar to that of Equation 1:
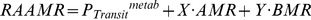
(3a)where



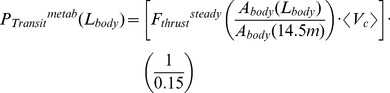
(3b)The value of the cross sectional area at 14.5 m (or *A_body_ (14.5* *m)* ) is *2.81* *m^2^* for both fin and blue whales, and follows from the allometry of *A_body_* shown in Tables 1, 2, 3, 4. The body fraction are set to *X = 0.15* and *Y = 0.2* but as with Equation 1, their specific choice yield small contributions in comparison to those of *P_transit_^metab^*. Note the efficiency factor of 0.15 discussed previously. Note also that *<V_c_> = *2.16 m s^−1^ and 2.4 m s^−1^ are applied at all body length for fin and blue whales respectively. As shown next, the RAAMR exceeds AMR by factors of only 1.1 to 1.6.

The metabolic rate incurred during prey-approach (PAMR) is calculated by assuming fluking thrust as being much greater than drag and (weight-adjusted) buoyancy (see figure 2 but without the engulfment-specific forces). With thrust being the only force at play, the (metabolic) power output of fluking is computed from the change in (whale) kinetic energy during a stage of duration *t_pa_*, namely from the end of the filter/recovery stage of the previous lunge to the beginning of engulfment of the current lunge. Factoring in the metabolic and fluke hydrodynamic efficiency, as well as the “rest-of-the-body” expenditures, one has:
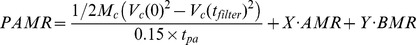
(4)


The fractions *X* and *Y* are set to *X = 0.25* and *Y = 0* as with EMR. Values for the whale’s mass (*M_c_*) and speed at the beginning of engulfment (*V_c_(0)*) can be found in Tables 1, 2, 3, 4. The values for *t_pa_* are shown Table 5 and extracted from tag studies [24], [25], [34], [35]. These correspond only to each species’ average body size. Finally, the speed at the end of the filter/recovery stage (*V_c_(t_filter_)*) is set at 1.0m /s for the three species, again as hinted by digital tag studies [24], [25], [34], [35].

**Table 2:** BLF3 simulation inputs – fin whales.

*L­_body_* (m)†	*L_0_* (m)†	*L_jaw_* (m)†	*w_head_* (m)†	*M_c_* (kg)†	*A_body_* (m^2^)†	*V_c_(0)* (m/s)†	*F_thrust_* (N)	*k_open_*
10.0	4.99	1.59	0.93	6853	1.02	1.45	1044	14.0
13.5	7.07	2.35	1.34	15 595	2.31	2.00	1852	11.7
17.7	9.68	3.32	1.86	32 758	4.84	2.63	3108	10.0
18.5	10.19	3.52	1.96	36 976	5.45	2.75	3382	9.7
20.2 (average length)	11.29	3.95	2.18	47 047	6.93	3.00	4000	9.1
22.7	12.92	4.59	2.51	64 770	9.52	3.37	4999	8.5
24.0	13.78	4.93	2.68	75 448	11.07	3.56	5560	8.2

† References 24, 39, 55–62.

Inputs applying to all body lengths: ρw = 1025 kg/m3, dt = 0.01 s, θgapemax = 78°, kam = 0.2, kopen/kclose = 1.82, CDopen = 0.5, CDclose = 0.5, CDbody = 0.05, Xjd = 1.2, Γ = 1.2, Vw(0) = 0 m/s, χ = 1.0, φ = 1.6 (0≤ t ≤0.66 topen), φ = 0.0 (0.66 topen < t ≤ tengulf). hsync is computed from Fsync/Xjd = (θgapemax/Γ) sin θgapemax Ljaw/(L0 - Ljaw) (Potvin et al [27]).

The symbols are further explained in the text, in Table 1, or in Text S1.

## Results

### Simulation Inputs

This BLF upgrade now requires, for each value of *L_body_*, a total of 22 inputs on body dimensions, body dynamics and hydrodynamics (Tables 1, 2, 3, 4). The former include the relevant body characteristics *L_body_*, *L_0_*, *w_head_*, *L_jaw_*, *M_c_* and *A_body_* (Figure 1) which were obtained from reduced major axis regressions [54] of morphometric studies [39], [55]–[62] in humpback, fin and blue whales. These regressions yield allometric equations of each body characteristic over body sizes ranging from weaned juveniles to the largest adults. Simulations were performed for minke whales as well, but at only one body size due to limited data.

As the most important body dynamics input, the initial speed of a whale just prior to the mouth opening stage (or *V_c_(0)* in Tables 1, 2, 3, 4) was determined from tag studies of large rorquals lunge-feeding at depth for krill [24]. These data, along with the modeling of possible escape scenarios of the prey [27], suggest this initial speed to vary as *V_c_(0) = V_n_ L_body_*, with *V_n_ = *0.159/sec (humpback), 0.148/sec (fin) and 0.146/sec (blue) [24]. Given the unavailability of tag data in the case of minke whales, the initial speed was assumed at *Vc(0)* = 1.16 m/s (as computed from *V_n_ = *0.15/sec), a value which turned out lower than the reported average minke cruising speeds (∼ 3 m/s [63]), but one that appears nevertheless within the range of this species’ feeding speeds.

**Table 3:** BLF3 simulation inputs – humpback whales.

L­_body_ (m)†	L_0_ (m)†	L_jaw_ (m)†	w_head_ (m)†	M_c_ (kg)†	A_body_ (m^2^)†	V_c_(0) (m/s)†	F_thrust_ (N)	k_open_
8.0	4.31	1.61	1.32	8000	1.97	1.27	1308	12.4
11.0	6.28	2.37	1.83	20 000	4.87	1.75	2405	10.8
12.7	7.45	2.82	2.13	27 869	7.32	2.02	3157	10.0
14.4 (average length)	8.64	3.28	2.42	46 226	10.46	2.29	4000	10.0
15.0	9.07	3.44	2.52	54 487	11.75	2.39	4319	10.0

† References 24, 39, 55–62.

Inputs applying to all body lengths: ρw = 1025 kg/m3, dt = 0.01 s, θgapemax = 78°, kam = 0.2, kopen/kclose = 1.26, CDopen = 0.5, CDclose = 0.5, CDbody = 0.05, Xjd = 1.03, Γ = 1.00, Vw(0) = 0 m/s, χ = 1.0, φ = 1.6 (0≤ t ≤0.66 topen), φ = 0.0 (0.66 topen < t ≤ tengulf). hsync is computed from Fsync/Xjd = (θgapemax/Γ) sin θgapemax Ljaw/(L0 - Ljaw) (Potvin et al [27]).

The symbols are further explained in the text, in Table 1, or in Text S1.

The values of the dynamical and hydrodynamic parameters *k_open_*, *φ, χ, C_D_* etc. listed in Table 1 are further discussed in *Modeling Details*. Note that with minke whales, and again due to lack of tag data, these (dimensionless) parameters were set at values typical of fin and blue whales given their similar morphology. An exception was with the maximum gape angle, which was set to 50° instead of 78°. BLF simulations, along with the film record, seem to suggest the impossibility for minke whales to carry out horizontal engulfments without premature cavity filling or draining at the maximum gape angle of 78° used by the larger Rorqual species (while lunging on krill).

### Body Motion, Engulfed Mass, Force Output and Expended Power

The simulation diagram sketched in Figure 3 yields calculations of the engulfed mass, muscular-based forces and whale body speeds, and ultimately of the energy expenditures. Sample outputs are shown below in the case of fin whales (outputs for humpback and blue whales are qualitatively similar). The scaling laws relevant to the graphed results are summarized in Tables 5, 6 and 7 for each species.

**Table 4:** BLF3 simulation inputs – minke whales.

L­_body_ (m)†	L_0_ (m)†	L_jaw_ (m)†	w_head_ (m)†	M_c_ (kg)†	A_body_ (m^2^)†	V_c_(0) (m/s)^††^	F_thrust_ (N)	k_open_
7.75 (typical adult length)	3.54	1.44	0.86	6650	1.02	1.16	641	18.4

† J. Goldbogen; unpublished data. ††Vc(0) = 0.15 Lbody,as with the large Rorquals of [24].

Other inputs: ρw = 1025 kg/m3, dt = 0.01 s, θgapemax = 50°, kam = 0.2, kopen/kclose = 1.49, CDopen = 0.5, CDclose = 0.5, CDbody = 0.05, Xjd = 1.00, Γ = 1.2 (same as fin whales), Vw(0) = 0 m/s, χ = 1.0, φ = 1.6 (0≤ t ≤0.66 topen), φ = 0.0 (0.66 topen < t ≤ tengulf). hsync is computed from Fsync/Xjd = (θgapemax/Γ) sin θgapemax Ljaw/(L0 - Ljaw) (Potvin et al [27]).

The symbols are further explained in the text, in Table 1, or in Text S1.

**Table 5:** Foraging durations from tag data.

	Humpback (14.0 m)	Fin (20.2m)	Blue (25.0m)	Data source
Prey-approach time (s)	8	12	18	J.A. Goldbogen; unpublished tag data
Engulfment time (s)	4.2	5.8	6.2	This paper and refs. [24], [34], [35]
Filter/recovery time (s)	13 (3)	28 (4)	55 (10)	Ref. [24]
Lunge duration (s)	41	53	98	Refs. [24], [34], [35]
Search time for prey in between lunges (s)	15.8	7.2	18.8	Data of the fourth row minus the sum of the first three rows
Maximum foraging dive duration (s)	11.3 (1.5)	9.3 (3.1)	11.6 (1.8)	Ref. [24]
Maximum number of lunges in a dive	12.3 (2.6)	6.5 (1.8)	4.3 (1.1)	Ref. [24]
Combined descent and ascent times (s), to depths approx 200 m (50 m)	180	186	246	Refs. [24], [34], [35]

The uncertainties are indicated in parentheses.

**Figure 4:**
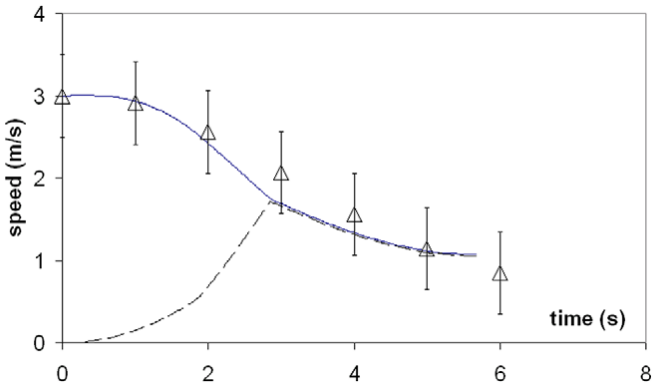
Theory meets experiment. Simulated speed of a 20.2 m fin whale (continuous line), as compared with data from digital tags [35] (triangles). The dashed line corresponds to the simulated speed of the engulfed mass. Note the *equivelocity* constraint operating during mouth closure (i.e., at *t >*2.85 s).

**Figure 5:**
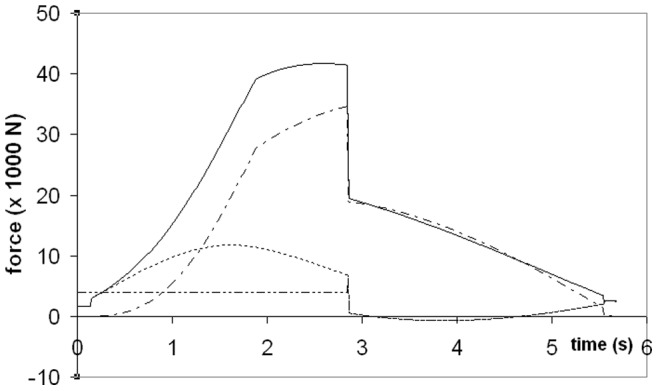
Simulated forces acting on a 20.2 m fin whale. Temporal variations of total drag (*F_SD_ + F_ED_*; continuous line), engulfment drag (*F_ED_*; dash-dotted), shape drag (*F_SD_*; dotted) and combination of fluking thrust and weight-adjusted buoyancy (*F_thrust_* + *F_ext_*; dash-doubly-dotted). Mouth closure begins at the 2.85 s mark and is characterized by shape drag cancelling *F_thrust_ + F_ext_* per equation 24.

Simulations of a 20.2 m fin whale are shown in Figures 4 and 5. These are compared in Figure 4 with averaged speed data collected by digital tags [35], as 20.2 m fin whales represent the average body size of this species [24]. The fact that the simulated lunges reproduced the measured speeds shouldn’t be too surprising given that several dynamic input parameters were tuned to yield a good match, in particular that of *F_thrust_* (at *t < t_open_*). Such parameter tuning was carried out only at the average body size of each species (where the tags data applies), and then scaled with respect to *L_body_* at all other body sizes according to scaling rules further discussed in *Modeling Details*.

The forces of engulfment are shown in Figure 5. Comparing with a similar figure produced in a previous version of the BLF shows overall qualitative agreement (see Diagram 10, in Potvin et al. [26] or Diagram 5 in Goldbogen et al. [25]). In this new version, however, engulfment drag is dominating over shape drag and fluking thrust during the entire engulfment sequence. Moreover, shape drag becomes very small as a result of the re-contact of the wake onto the whale’s decelerating body. In fact, shape drag can become negative at sufficiently large body size, i.e., a pushing force rather than a resistance (as illustrated further in *Modeling Details*), in analogy with the wake pushing a power boat upon engine cut-off. But here the effects of wake re-contact on the forward motion are mitigated by fluke thrust which then becomes negative, i.e., when producing a braking rather than a propulsive action. Interestingly, such wake re-contact affects only the larger sized whales given their greater decelerations, a result of having to push forward a larger engulfed mass (relative to body size) as caused by the positive allometry of the skull (more on this below).

**Figure 6:**
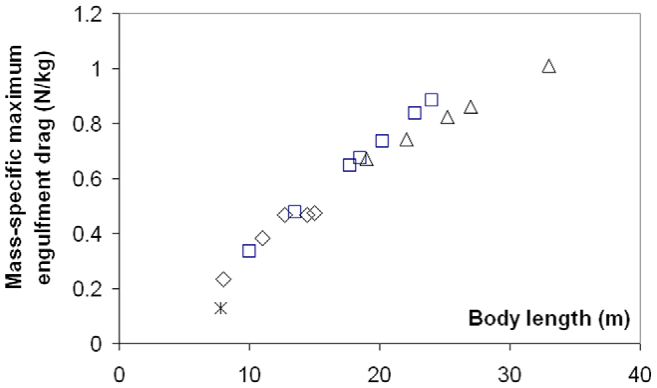
Maximum engulfment drag (mass-specific). Largest value attained during mouth opening, by humpback (diamonds), fin (squares), blue (triangles) and minke whales (starburst).

**Figure 7:**
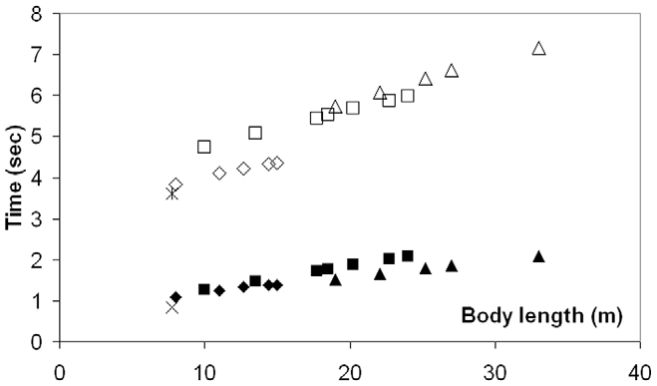
The time scales of engulfment drag. Engulfment time (*t_engulf_*; empty symbols) and VGB push time scale (*τ*; filled symbols) for humpback (diamonds), fin (squares), blue (triangles) and minke whales (starburst and times).

The variations in intra- and inter- specific scaling among the humpback, fin and blue whales can be summarized by the (mass-specific) peak engulfment drag (i.e., *F_ED_* at *t = t_engulf_/2* (the time of maximum gape)) and engulfment time *t_engulf_* shown in Figures 6 and 7 (see also Table 6). Note that Figure 7 also shows the so-called VGB contraction time *scale τ* (in contrast to engulfment *duration*), which is about three times as small, and in the range of 1 to 2 seconds over the entire body dimensions of humpback, fin and blue whales, as further discussed in *Modeling Details*.

**Table 6:** Scaling of the buccal cavity wall force and engulfment times with respect to body length (*L_body_*).

Symbol	Morphological Parameter	Species	n	Slope	Coefficient	R^2^
*F_BC_^max^/M_c_*	Specific peak cavity wall force (N/kg)	Humpback whale	5	1.142	0.0231	0.93
*F_BC_^max^/M_c_*	Specific peak cavity wall force (N/kg)	Fin whale	7	1.099	0.0271	0.99
*F_BC_^max^/M_c_*	Specific peak cavity wall force (N/kg)	Blue whale	5	0.745	0.0743	0.99
*t_engulf_*	Engulfment duration (sec); Eqn. 3	Humpback whale	5	0.204	2.507	0.99
*t_engulf_*	Engulfment duration (sec); Eqn. 3	Fin whale	7	0.269	2.526	0.99
*t_engulf_*	Engulfment duration (sec); Eqn. 3	Blue whale	5	0.408	1.721	0.99
*Τ*	VGB push time scale (sec); *t_engulf_/√k_open_*	Humpback whale	5	0.384	0.494	0.98
*Τ*	VGB push time scale (sec); *t_engulf_/√k_open_*	Fin whale	7	0.574	0.334	0.99
*Τ*	VGB push time scale (sec); *t_engulf_/√k_open_*	Blue whale	5	0.583	0.271	0.99

### Metabolic Expenditures

**Figure 8:**
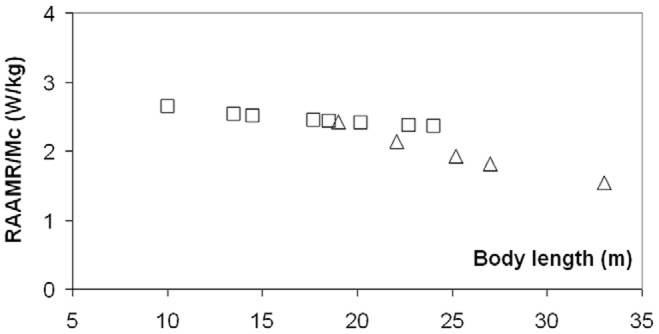
Mass-specific Rorqual Average Active Metabolic Rate (RAAMR), calculated for fin (squares) and blue whales (triangles).

**Figure 9:**
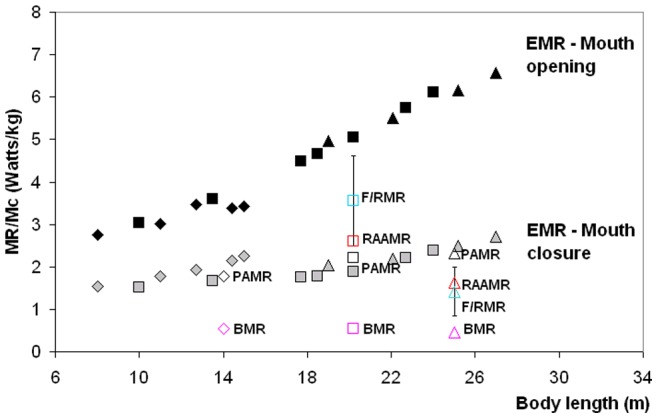
Mass-specific Metabolic Rates (MR) across scale. Ratios calculated for humpback (diamonds), fin (squares) and blue whales (triangles). Engulfment Metabolic Rate (EMR) during mouth opening – black symbols, and during mouth closing – gray symbols. Metabolic rates for prey-approach (PAMR), basal (BMR), filter/recovery (F/RMR) and Rorqual Average Active (RAAMR) – open symbols as indicated.

Figures 8 and 9 show the calculated metabolic expenditures (mass-specific) during non-feeding transport (RAAMR; Figure 8) and in the course of lunge-feeding (EMR; Figure 9). Unlike the metabolic expenditures of engulfment, RAAMR decreases slightly with body size from about 2.7 to 1.7 W kg^−1^ (see also Tables 7 and 8). Thus, RAAMR is consistent with the general concept of a lower cost of transport [43], although we note that RAAMR is somewhat larger than AMR (equation 2) by factors of 1.1 to 1.6. As calculated and applied to averaged sized fin and blue whales, RAAMR is greater than BMR by factors of 4.8 and 3.6 respectively.

**Table 7:** Scaling of the metabolic power (EMR or RAMMR) expended during engulfment (mouth opening only) and non-feeding swimming, with respect to body length (*L_body_*).

Symbol	Mass-specific power ratio (W/kg)	Species	n	Slope	Coefficient	R^2^
*EMR|_mouth open_ /M_c_*	Specific expended power - mouth open	Humpback whale	5	0.370	1.277	0.88
*EMR|_mouth open_ /M_c_*	Specific expended power - mouth open	Fin whale	7	0.803	0.458	0.98
*EMR|_mouth open_ /M_c_*	Specific expended power - mouth open	Blue whale	5	0.794	0.476	0.99
RAMMR */M_c_*	Specific expended power - non-feeding travel	Fin whale	8	^−^0.130	3.558	0.99
RAMMR*/M_c_*	Specific expended power - non-feeding travel	Blue whale	5	^−^0.809	26.23	0.99

In contrast, the estimation of the EMR increases with body size as demonstrated by previous studies [24]. But these simulations show for the first time that metabolic output is highest during the mouth opening stage when the cavity wall forces are at their peak (Figure 5). Overall, such intensity ranges from 3 to 7 W kg^−1^, where the largest expenditures are incurred at the largest body sizes. This is a reflection of the positive allometry of the skull which enhances buccal cavity volume (relative to body size) and results in larger mass-specific engulfed and deflected water masses [23].

Figure 9 also compares the metabolic rates involved in each phase of a lunge, namely, prey-approach (PAMR), mouth opening and closure (EMR) and filter/recovery (F/RMR). Although PAMR and EMR are calculated directly in terms of the forces involved (see Eqs. 1, 2, 4), F/RMR is estimated only indirectly, i.e., via PAMR, EMR and RAAMR (Equation 3), and from the average metabolic output (*ΔE_O2_/t_maxdive_*) that could be obtained from a “maximum duration” foraging dive in which all of the stored oxygen would (theoretically) be used to achieve a maximum number (*f_maxlunge_*) of lunges:
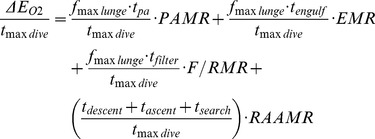
(5)


Parameters *t_pa_*, *t_engulf_* and *t_filter_* correspond to the duration of the prey-approach, engulfment, filter/recovery stages respectively, and *t_ascent_*, *t_decent_*, *t_search_* and *t_maxdive_* to the times needed for descending and ascending to and from foraging depth, searching for the krill patch in between lunges (during a same dive), and for performing the longest dive possible with the O_2_ stores at hand (O_2_ stored in the lungs, muscle and blood). With the exception of *t_engulf_*, most of these parameters are obtained from tag data and listed at the representative average body length of each species in Table 5. The physiological data pertinent to the computation of *ΔE_O2_* are supplied in Table 9. This estimate of the F/RMR is similar to a TADL calculation [24], [28], [29] except that *t_maxdive_* is not predicted but rather used as an input to yield a metabolic rate. This is a rather speculative estimate given that several input parameters are still poorly known. Nevertheless, the results suggest that the metabolic requirements of engulfment (mouth opening specifically) significantly exceeds those of the other stages of a lunge by 100% or more. Moreover, both PAMR and F/RMR still represent “active” metabolic outputs (in contrast to “basal”), as they turn out to be similar to those of RAAMR. This shouldn’t be too surprising even for the filter/recovery phase, as the significant oxygen deficits incurred during mouth opening stage are likely to require high recovery metabolism.

## Discussion

Body size is one of the most important determinants of energetic efficiency and locomotor performance [64]–[70]. Because locomotion and prey capture are integrated in rorquals, as they are in many vertebrate taxa [71], both elements are subject to mechanical scaling effects. Here we integrated morphological and kinematic data into a novel hydro-mechanical model, derived from first principles and from a unified theory of predator-prey dynamics [26], to estimate the forces required for lunge feeding whales. The model output enabled us to quantify the energetics of feeding across an extensive size range of juvenile and adult rorquals. In general, the energy and power output required to lunge feed increases disproportionally with increasing body size, a phenomenon that results from a complex interaction between lunge speed, unsteady hydrodynamics and the allometric scaling of the engulfment apparatus [23]. Because feeding is such a major component of baleen whale life history, these predictions have major consequences for rorqual foraging ecology, ontogeny, and evolution.

### The Metabolic Cost of Engulfment

**Figure 10:**
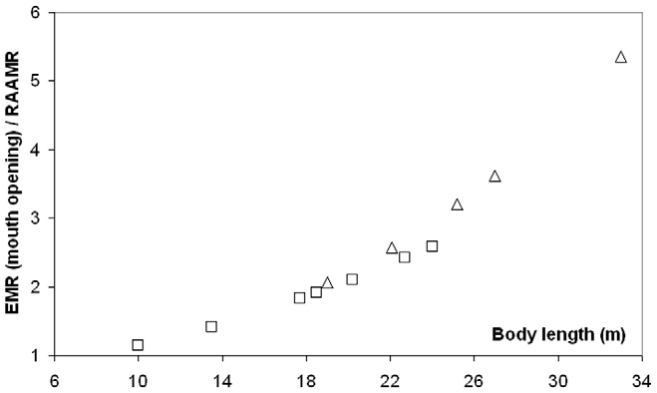
Mouth opening EMR as compared to RAAMR. Ratios calculated for fin (squares) and blue whales (triangles). The 33m blue whale is non-extant.

The results of Figure 9 show how significantly more strenuous engulfment is during mouth opening, in comparison with BMR and non-feeding transport. This is emphasized further in figure 10, with a direct comparison with RAAMR at all body sizes in fin and blue whales. Unlike engulfment metabolic expenditures, RAAMR (mass-specific) decreases slightly with body size (Figure 8; see also Tables 7 and 8). This is similar to the Active Metabolic Rate (AMR) of other marine mammals which is approximately three times their Basal Metabolic Rate [41], [72]–[74]. But the ratio of EMR to RAAMR, much like all other ratios that relate engulfment costs to all other energetic expenditures, increase with body size for all three species up to about 3.7 RAAMR (Figure 10). Most noteworthy is the convergence of EMR and RAAMR at small body sizes, and the conclusion that the metabolic expenditures during engulfment become quite close to those of non-feeding swimming at such sizes. At large body sizes on the other hand, and including the sizes of the non-extant blue whale (33 m), the much greater costs of engulfment become evident.

**Table 8:** Scaling with respect to body length (*L_body_)* of the expended metabolic power ratios during engulfment (EMR; mouth opening only), as compared with AMR, RAAMR and MMR.

Symbol	Power ratio	Species	n	Slope	Coefficient	R^2^
*EMR|_mouth open_ /AMR*	Specific expended power (mouth open ) over AMR	Humpback whale	5	1.120	0.104	0.99
*EMR|_mouth open_ /AMR*	Specific expended power (mouth open ) over AMR	Fin whale	7	1.148	0.035	0.99
*EMR|_mouth open_ /AMR*	Specific expended power (mouth open ) over AMR	Blue whale	5	1.785	0.014	0.99
*EMR|_mouth open_ /RAAMR*	Specific expended power (mouth open ) over RAAMR	Fin whale	7	0.933	0.128	0.98
*EMR|_mouth open_ /RAAMR*	Specific expended power (mouth open ) over RAAMR	Blue whale	5	1.730	0.012	0.99
*EMR|_mouth open_ /MMR*	Specific expended power (mouth open ) over MMR	Humpback whale	5	0.760	0.0461	0.97
*EMR|_mouth open_ /MMR*	Specific expended power (mouth open ) over MMR	Fin whale	7	1.160	0.0161	0.99
*EMR|_mouth open_ /MMR*	Specific expended power (mouth open ) over MMR	Blue whale	5	1.369	0.0085	0.99

**Table 9:** Values used in the calculation of the Filter/Recovery Metabolic rate (F/RMR; equation 5).

Parameter	Symbol	Unit	Computational	Blue	Fin	Humpback	Reference
**(1) Morphology**							
Body length	L_body_	m		25	20	14	[39]
Body mass	M_c_	kg		92,671	52,584	35,692	[39]
Body volume	U_c_	m^3^		86.4	45.9	31.5	
Body surface area	A_body_	m^2^	SA = 0.08M_c_^0^.^65^	131.5	87.1	68.2	[14]
Fluke total surface area (m^2^)	A_fluke_	m^2^		9.19	6.26	10.43	[14] [24]
**(2) Oxygen stores**							
*O_2_ stores (Lungs)*							
Total lunge capacity	TLC	l	0.1 X M_c_^0^.^96^	5865	3404	2347	Kooyman, 1989
Diving lunge volume	DLV	l	0.75 X TLC	4399	2553	1760	Goforth, 1986
Total O_2_ in lungs	O_lung_	l	0.15 X DLV	660	383	264	Kooyman, 1989
*O_2_ stores (muscle)*							
Muscle mass	M_m_	%M_c_		39.4	45.6	30.6	[39]
Muscle mass	M_m_	kg		36,512	23,978	10,922	
Myoglobin	M_b_	g	34 g kg^−1^	1,241,421	815,262	371,340	Noren & Williams, 2000
O_2_ combining capacity	η_O_	1 g^−1^ Mb		0.0013	0.0013	0.0013	Kooyman, 1989
Total O_2_ in muscle	O_muscle_	l		1614	1060	483	
*O_2_ stores (Blood)*							
Blood volume	BV	l	0.127 l kg-1	11,769	6,678	4,533	Ridgway et al., 1984
Arterial volume	AV	l	0.33BV	3,884	2,204	1,496	Lenfant et al., 1970
Venous volume	VV	l	0.67BV	7,885	4,474	3,037	Lenfant et al., 1970
Arterial haemoglobin	AHB	g	209 g l^−1^	811,723	460,593	312,633	Ridgway et al., 1984
Venous haemoglobin	VHB	g	209 g l^−1^	1,648,043	935,144	634,740	Ridgway et al., 1984
O_2_ combining capacity	η_O_	1 g^−1^ Mb		0.0013	0.0013	0.0013	
arterial blood O_2_	ABO	l	98% saturation	1034	587	398	Kooyman, 1989
venous blood O_2_	VBO	l	75% saturation	1656	940	638	Kooyman, 1989
Total O_2_ in blood	O_blood_	l	ABO + VBO	2690	1527	1036	
							
Total Body O_2_ stores	O_body_	l	O_lung_ + O_muscle_ + O_blood_	4964	2969	1783	
Liters/min burned at BMR rate		l O_2_/min	O_body_/4.0M_c_^0^.^74^	2.98	2.98	2.98	[24]

**Figure 11:**
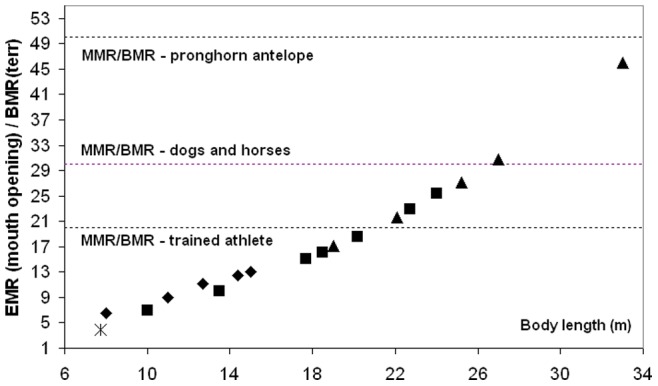
Mouth opening EMR as compared to the Basal Metabolic Rate of terrestrial mammals. Ratios calculated for humpback (diamonds), fin (squares), blue (triangles) and minke whales (starburst). Note that the 33 m blue whale is non-extant.

**Figure 12:**
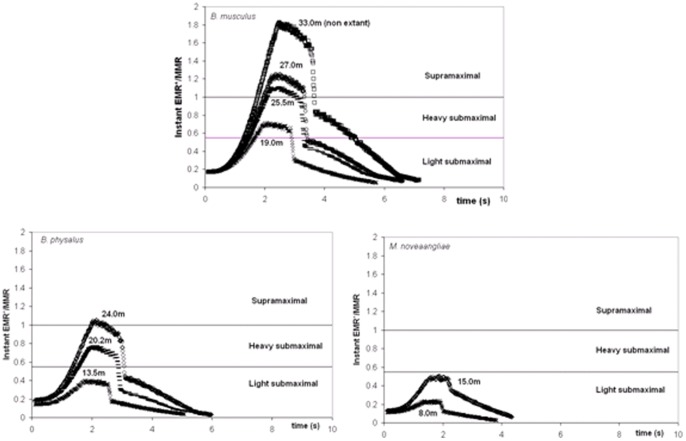
Comparing instant mouth opening EMR* with MMR. EMR* corresponds to equation 1 with the energies integrated over time slices of 0.1 s durations, but without the X- and Y-terms.

Engulfment metabolism relative to other standard metrics of power is shown in Figure 11, with a comparison of EMR with the BMR and maximal metabolic rate (MMR) of terrestrial mammals of the same mass (MMR data from [75]; and *BMR(terr) = 4.0 M_c_^0^.^75^* (in watts)). Marine mammals are different from terrestrial mammals, of course, with their heightened resting metabolic rate on the one hand [41], and their well-known physiological adaptations for overall BMR reduction during diving on the other (for example, bradycardia and regional vasoconstriction) [31]. But the data available on mitochondrial volume densities [76] and aerobic scope [77] on seals and dolphins already point to a similar aerobic metabolic performance for powering locomotor musculature (even during breath-hold), which after all, is the main function of metabolism during strenuous exercise [68]. Thus the physiological demands of lunge-feeding rival those of athletic [75], or more appropriately “highly active”, terrestrial mammals during maximal effort. What is also interesting is the finding that the relative level of power required by engulfment increases significantly with body size, from that similar to trained human athletes at the smaller sizes (MMR ∼ 20 BMR), to the maximum performance of dogs and horses at the larger sizes (MMR ∼ 30 BMR). Although interesting, this comparison actually underestimates the actual maximum metabolic output that may be required, as EMR represents an averaged metabolic rate, i.e., over the duration of mouth opening. A more informative datum is the “instantaneous EMR” (or EMR*) computed from averaging power output over the significantly shorter time interval of 0.1s, to become time-dependent as shown in Figure 12 (Note: EMR* also neglects the *X-* and *Y-*terms using in equation 1). EMR* indeed yields significantly higher maximum values, i.e., EMR*|_max_/BMR(terr) = 5.8 and 18.2, 12.6 and 39.8, and 24.3 and 48.2, at the smallest and largest sizes in humpback, fin and (extant) blue whales respectively. Instant EMR thus changes the effort picture of Figure 11 somewhat, suggesting the largest extant Rorquals as engulfing prey and water at performance levels rivaling those of the pronghorn antelope. Interestingly, the predicted metabolic performance required from the non-extant 33 m blue whale far exceeds that of the pronghorn antelope at EMR*|_max_/BMR(terr) = 78.6.

Maximum aerobic capacity
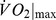
is assessed with a comparison of instant metabolic rate with the *Maximum Metabolic Rate* (i.e., *MMR = 39.4 M_c_^0^.^87^* (in watts [75])), as listed in Table 8 and shown in Figure 12. The figure shows the metabolic demands of engulfment in relation to MMR, which is a direct measure of the maximum aerobic capacity 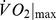
of an air-breathing terrestrial mammals [68], or in other words, of the limitation of oxidative metabolism of muscle cells to supply energy without recourse to anaerobic glycolysis [68]. Exceeding MMR as shown reveals the magnitude of the oxygen deficit that accumulates during the most demanding phase of engulfment. But oxygen deficits also accumulate from the start, i.e., at small effort level, given the short time scales of engulfment, and also for fueling the anaerobic metabolic reactions that are needed to power the forward push of the engulfed mass by the fast twitch muscle fibers embedded in the VGB (such fibers represent approximately 50% of the VGB muscle fibers [R.E. Shadwick, unpublished data]). Accordingly, overall oxygen deficits may be small and recovered during the latter part of the effort, as during typical *light submaximal exercise* [31]; or greater, i.e., as during *heavy submaximal* exercise, and mostly repaid during longer post-effort recovery (i.e., during filtering or after a dive); or significantly greater (*supramaximal exercise*), where severe muscle fatigue induced by stored glycogen depletion and inorganic phosphates accumulation can only be recovered during extended rest periods. Again, the comparison suggests that engulfment is disproportionally costly for larger body sizes. At the upper extreme of body mass in the (extant) range of 25 to 27 m, the rates of energy expenditure are most demanding, i.e., reaching MMR-levels over several seconds.

### Scaling of Lunge Feeding Power Output: Consequences and Implications

Our analyses show again that the energetic cost of engulfment is positively allometric whereby larger rorquals must expend relatively more energy to lunge feed [23], [24], [27]. But the results show for the first time that it is the rate of energy delivery (i.e., the power), rather than the total energy required for lunge feeding, that may be the limiting factor on lunge performance. This is underscored on the one hand by the TADL computed from our simulated energies (and physiology data of [24]), hinting at maximum dive times in the range of 9 to 12 minutes at all body sizes, including that of the non-extant 33 m blue whale; and on the other, by the power requirements reaching levels of high muscle fatigue and long post-dive recovery. Such a heightened power requirement is a result of the positive allometry of the engulfment apparatus [23], where larger skull sizes (in relation to body size) yield greater engulfment capacity and increased captured mass kinetic energy costs, *as performed over (nearly) unchanging engulfment time scales brought about by the evasion strategies of the prey* [27].

Increased fatigue and metabolic recovery may explain the reduction in lunge frequency seen across species [24], and presumably, among the largest individuals of each species. However, other factors such as krill patch dimensions may also be an important limiting factor, particularly if they are similar to, or smaller than predator size. But the noted increased post-dive breath numbers in humpback whales after dives encompassing the largest number of lunges [34] may provide supporting evidence for intensified power delivery rather than for food availability. Interestingly, such ability to deliver the required power may be adversely affected for emaciated whales returning to their feedings grounds after months of fasting, and as a result, experience lower lunge frequencies than at their normal body weight. This is an interesting topic for future simulations of course, but one that will be possible only when the morphology and body mass of these underweight and slimmer animals become known.

Limits on power delivery predictably has a negative impact on filtering capacity over an entire dive [24], and therefore feeding efficiency [25], but such power demands of engulfment may also set a hard physiological limit on maximum body size in rorquals. We tested this hypothesis by simulating lunge feeding in a hypothetical 33 m blue whale, and the results indicate that its engulfment power expenditures exceed those of MMR by over 80%, with a peak instant EMR of about 78.6 BMR(terr). In other words, blue whales at this scale that would be able to generate the needed power for engulfment would do so at a cost of requiring extensive rest and recovery even after a single lunge, to obviously limit residence time in high density prey patches at depth. In this way, and as argued for dinosaurs [78] and blue whales [23], limits in body size may not only be imposed by a balance between resource availability and energy expenditures, but also by the metabolic power output required for feeding (see also this argument applied to non-feeding transport in [52]). Nevertheless, either circumstance (power vs. energy limitation) predictably constrains maximum body size in this lineage of baleen whales. However, it is unknown what limits size in other cetacean lineages, as well as other lineages of other aquatic tetrapods that also exhibited gigantism in the past [67]. Clearly more research is needed to explore what factors limit body size in different taxa not only from an energetics perspective, but also with respect to other life history constraints.

Our analyses, including those of the minke whale (a fish- as well as a krill-feeder), also have implications for the lowest body size classes of rorquals, which suggest relatively low power requirements for lunge feeding. This has a significant impact on the ontogeny of rorquals since weaned juveniles reap the advantages of efficiency in a low-cost feeding strategy that will facilitate rapid growth. Such a characteristic, which is exemplified by the large range in intraspecific body size of extant rorqual species, likely played a major role in the evolution of gigantism in balaenopterids. Lower energetic costs associated with engulfment at smaller body sizes may also have implications for how lunge feeding evolved in smaller ancestral baleen whales. Fossil evidence between the late Oligocene and late Miocene indicate that extinct rorquals reached maximum body sizes no larger than extant minke whales (i.e. 10 m long) [79]. These data, together with our minke results (Figure 11), suggest that lunge feeding evolved within a body size range where the cost of engulfment does not appear to be significantly higher than non-feeding swimming (Interestingly, the minke modeling suggests that such costs may be even smaller). We posit that the evolution of baleen in cetaceans [80] at small body size classes [79] generated a mode of feeding that exhibited high energetic efficiency where vast amounts of prey could be captured at a relatively low cost [25]. Such an ecological role further led to more specialized types of microphagy, and therefore to several mysticete lineages that each exhibited distinct filter feeding modes, that satisfied particular niches related to differences in prey type. The ability to gulp discrete volumes of prey-laden water likely facilitated the exploitation of more agile zooplankton (i.e. krill, squid, etc.), in contrast to slow moving copepod prey for which continuous ram feeding may be more efficient [5], and therefore promoted the evolution of extremely large rorqual species fueled by the existence of super-aggregations of krill [81]. Future research should further explore the feeding energetics of the smallest baleen whale species, both extinct and extant, to examine how different feeding modes may have evolved and to what extent it promoted extreme body size.

### Conclusions

This paper has shown that the physical requirements of lunge-feeding towards krill, particularly with regards to the rate of energy delivery during engulfment, may present an obstacle to ever increasing body size. This would follow from the allometry of the skull, along with the approach speeds being dictated by the evasion strategies of the prey. But this obstacle may, of course, be altogether avoided if lunges can be performed differently, for example by reducing the maximum gape angle, or by passively engulfing while lunging vertically and/or cooperatively, as already performed by some rorqual species lunging at the surface. Furthermore, limits to body size are also connected to prey type and availability. Large body size obviously limits maneuverability and has most likely forced the blue whale, the largest of the Rorquals, into obligate krill-feeding and into exploiting a resource that has so far been abundant enough to even support these giants in the very large numbers of pre-whaling days. But ultimately, and given that most Rorquals prey on both krill and fish, the linkages between food resources, motion energetics, and body size will not be completely resolved until further modeling is carried out on lunge-feeding towards fish. In general, lunging on fish will demand significantly higher prey approach speeds and accelerations by the predator. In most rorqual species such extra effort will be mitigated by the use of smaller gape angles (as with minke whales), as well as by other maneuvering and dynamic adaptations such as vertical lunging without active VGB muscle action. Although vertical lunging at higher speeds and with smaller gapes can already be simulated with the modeling techniques discussed here, passive engulfment is bound to change the physics and hydrodynamics of the modeled whale-ocean system, and as a result, yield an alternate simulation tool for the study of lunge feeding.

### Modeling Details

#### Body and fluid dynamics constraints from s*ynchronized engulfment*

Synchronized Engulfment introduces several constraints on overall engulfed mass motion as well as on specific dynamic variables. The first concerns the eccentric contraction of the VGB musculature, which must impart forward motion to the engulfed mass (as a *reflux*) without *premature draining* of the cavity [26]. Here premature cavity draining occurs whenever the engulfed water leaves the mouth aperture prior to complete mouth closure. Moreover, the BLF implements an assumption of *premature filling avoidance*, or in other words, of preventing complete cavity filling post-TMJ prior to maximum gape, and complete cavity filling ant-TMJ prior to mouth closure. These two constraints are implemented by the use of cavity wall force pushing the engulfed mass with the “right” amount: If the whale exerts a force that is too high, the result is a more rapid reflux, slower whale motions and thus a slower cavity inflation rate or even a negative inflation rate (or draining). Conversely, if the force is too small the reflux is minimized, but the whale speed remains high and the ventral pouch fills too quickly. Note that an extreme of the latter includes “passive” or “compliant” engulfment [19], where the influx of water is met with little resistance other than the passive mechanical (i.e., elastic) properties of the VGB. In this case, and given the compliant nature of the VGB over most of its allowed strain range [19], little force is exerted on the engulfed mass during most of the engulfment process.

Because the oropharyngeal cavity (post-TMJ) does not significantly fill during mouth closure, the model invokes a third SE-motivated constraint, namely that of the engulfed mass post-TMJ moving at the instantaneous speed of the whale. This so-called *equivelocity* constraint is new to the BLF and is further extended to the water captured anterior to the TMJ, as motivated by the water being “scooped” by the rotating mandibles during mouth closure rather than “bagged-in” and pushed forward as during the mouth-opening stage. Most importantly, equivelocity implies the absence of significant surging of engulfed water moments before complete closure, a state confirmed by the film record. Equivelocity, and its consequent *equiacceleration*, means that the interaction between whale and water represents a perfectly inelastic collision, and leads to a useful derivation of an engulfment hydrodynamic force that would otherwise be very difficult to determine (i.e., *F_ww_* in Figure 2). Furthermore, equivelocity provides an additional constraint on drag and thrust and as such helps reduce the uncertainties that have plagued the modeling of previous studies [25]–[27]. The equivelocity of whale and engulfed fluid motions during mouth closure, as well as the acceleration of the *reflux* during mouth-opening, are clearly visible in Figure 4. (Note that there is no reflux in passive engulfment [19], [26]).

#### Algorithmic flow of the BLF

The hydrodynamic model is an iterative scheme aimed at computing the forces applied to, and speeds sustained by both whale and engulfed mass. As shown in the flow chart of Figure 3, each iteration involves a calculation of the gape angle, mouth surface area (projected longitudinally) and mass so-far engulfed, which are then used in the computation of all the forces acting on the whale’s body and engulfed mass. The upgrades of this BLF version include the use of engulfed mass rates that are specific to the mouth opening and closing stages – an important ingredient for ensuring physical realism at the needed time scale of metabolic output; other new features implement a formulation of shape drag aimed at approximating the effects of wake re-contact on the whale’s body during mouth closure, and the use of the *equivelocity* constraint to derive the “ocean-to-engulfed mass” drag *F_ww_*, a force that is entirely dynamical in nature.

#### Mouth opening rates and engulfment duration

The complete gape angle cycle of engulfment, from opening to closure, lasts several seconds, with the mandibles opening to a maximum gape (*θ_gape_^max^*) of about 78–80° with the humpback, fin and blue whales [17], [18], and of about 50° with the minke whale [JAG unpublished data]. Evidence from the film record suggests rates of mouth opening and closure calculated as [27]:
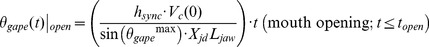
(6)

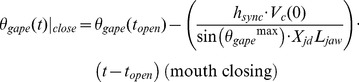
(7)with *θ_gape_* and *t* corresponding to the gape angle (Figure 1) and time respectively. Here *L_jaw_, X_jd_, h_sync_, t_open_* and *V_c_(0)* are the length of the mandibles, jaw disarticulation factor, synchronization factor, duration of the mouth-opening stage and whale’s speed just prior to mouth opening, respectively (Figure 1).

The body dimensions used in these formulae (and everywhere else in the BLF) are shown in Tables 1, 2, 3, 4 and characterize the size classes typical of adults in all three Rorqual species [24]. These are the results of reduced major axis regressions [54] of morphometric studies [39], [55]–[62]. The initial whale speeds *V_c_(0)* at these average body sizes were obtained from tag data [24]. The duration of mouth opening (*t_open_*) follows from the requirement of the (near) maximal extension of the ventral pouch post-TMJ by the time of maximum gape [27]. On the other hand, mouth closure duration is assumed as being the same as mouth opening, as motivated from video footage [22]. When integrated with Equations 6 and 7, this observation yields the means of calculating the durations of engulfment (*t_engulf_*), mouth opening (*t_open_*) and mouth closure via.
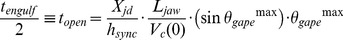
(8)


The ratio *h_sync_*/*X_jd_* can be shown to scale as *h_sync_*/*X_jd_ = (θ_gape_^max^/Γ) sin θ_gape_^max^ L_jaw_/(L_0_ - L_jaw_)* as a direct result of Synchronized Engulfment [27], with *L_0_* representing the axial length of the VGB (i.e., from umbilicus to mandibular symphisis) (Tables 1, 2, 3, 4). The value of constant (input) *Γ* listed in Tables 1, 2, 3, 4 is derived from the values of the ratio *h_sync_*/*X_jd_* extracted from the temporal development of the gape angle shown in video footage [27].

Equation 8 is important as it *predicts* the duration of engulfment at all body sizes. It also provides an important scale for the expression of the force provided by VGB musculature (see equations 16 and 17 below). Engulfment duration is the first of two fundamental time scales that are relevant to engulfment, as further discussed in the context of cavity wall force (*F_BC_*).

#### Engulfed mass capacity

The overall capacity of the ventral pouch, as well as the filling rate of each compartment (i.e. ant-TMJ and post-TMJ), are important dynamical components of the model. These volumetric capacities are expressed in terms of quarter-ellipsoidal shapes [23]. When filled during typical horizontal lunges, and excluding local cavity over-extension effects due to sloshing, photogrammetric data suggest the ventral pouch’s extensibility to not expand in width beyond that of the skull and in depth below the length of the mandibles (at maximum gape). From these observations emerge the following “filled” capacity equations:
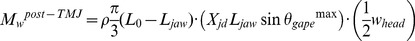
(9)

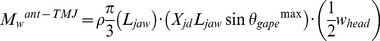
(10)


Parameters *ρ_w_* and *w_head_* correspond to the density of sea water and width of the skull (Figure 1; Tables 1, 2, 3, 4). Like all other body dimensions, the latter is known from morphometric studies. The quantities in parentheses correspond to the three semi-minor radii of the quarter-ellipsoids. Interestingly, the ratio of these capacities amounts to *M_w_^ant-TMJ^*/*M_w_^post-TMJ^* ∼ 0.5 for all three sizes of the adult rorqual species represented in Tables 1, 2, 3. Note also that Equations 9 and 10 would not apply to the cavity of bloated dead whales for which the width well-exceeds that of the skull, a result of the decomposition gases stretching the VGB to longitudinal and circumferential strains never reached during actual lunge feeding.

Using the morphological data of Tables 1, 2, 3, 4 in the ratio of engulfed mass to body mass *M_w_^total^/M_c_* (with *M_w_^total^* ≡ *M_w_^post-TMJ^ + M_w_^ant-^* ^TMJ^) shows engulfment capacity to exceed body mass by at least 10% at most body sizes, and up to 50% at the largest body sizes (Goldbogen et al [25]). Such large engulfment volumes are enabled by the mouth opening at wide gape angles, along with the unfolding and stretching of ventral pleats that line the buccal cavity wall. Those pleats and the rest of the VGB are reversibly extensible up to several-times its resting length [19]. The volume of the cavity is also increased by the inversion and distension of a weakly-muscularized and highly elastic tongue (see Diagram 6 in Lambertsen [8] or Diagram 3 in Goldbogen [9]), which invades an intermuscular space (the so-called the *cavum ventrale*) located between the VGB and the rest of the body [8], [82], [83]. The result is that at maximum cavity extension, the engulfed water mass runs ventral and posterior to the esophagus [9].

#### Engulfment rates

During mouth opening the filling rate of the cavity post-TMJ, or in other words the amount of fluid mass *M_w_(t)* entering per unit time (or *dM_w_/dt* 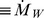), is seen as the filling of a quarter-ellipsoidal sac opened at its wide end (see Diagram 2 in [23]). The opening of the latter is assumed to be shaped as a half-ellipse of surface area a given by:
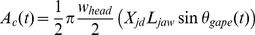
(11)


(see also Diagrams 5a,b,c in Potvin et al [26]). This instantaneous area is known once the gape angle is calculated from equation 6 or 7. The water flows into the ventral pouch at a speed *φ*(*V_c_(t)* – *V_w_(t))*, resulting in the flux being given by:
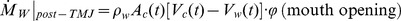
(12a)


Here the velocities *V_c_* and *V_w_* are obtained from the equations of motion to be discussed next. Parameter *φ* is a filling efficiency necessitated by the water entering the cavity (below the TMJ) at speeds that are, relative to the whale, higher than the speed of the ocean ahead, a result of the flow passing through a funnel formed by the palate and buccal cavity ant-TMJ. Specific values were obtained via numerical experimentation constrained to not exceed the volume of equation 9, resulting in *φ = 1.60* for *t ≤0.66 t_open_* and *φ = 0* for *0.66 t_open_ < t ≤ t_open_* (in all three rorqual species). The latter condition, which effectively stops the filling of the cavity 66% into mouth opening duration was necessary for avoiding cavity draining. This constraint leads to the cavity post-TMJ to fill to somewhere between 70% and 80% of maximum engulfment capacity.

The filling rate of the cavity ant-TMJ during mouth closure is based on a rate of (upward) angular sweep corresponding to the motion of the mandibles:
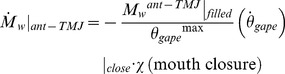
(12b)where the *θ-dot* derivative (or *dθ/dt*) on the right-hand-side is obtained from Equation 7. Parameter *χ* represents a capture efficiency quantifying possible fluid loss during mouth closure (*χ = 1* here). Note that equation 12b was not implemented in the previous version of the BLF [26], thus omitting the filling of the buccal cavity ant-TMJ. This omission was partially compensated by letting the cavity post-TMJ fill to 100% capacity throughout engulfment, but still resulted into a 25% underestimate in the computation of the engulfment forces (see Appendix 1 of Goldbogen et al. [25]).

The instantaneous value of the total mass accumulated so far (*M_w_^total^*) is obtained from a numerical integration of Equation 12a during mouth opening, and of Equation 12b during mouth closure:

- Mouth opening (0≤ *t* ≤ *t*_*open*_ = *t*_*engulf*_/2).
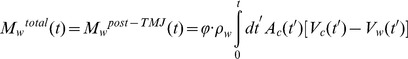
(13a)


- Mouth closure (*t*_*open*_ < *t* ≤ *t*_*close*_ = *t*_*engulf*_).
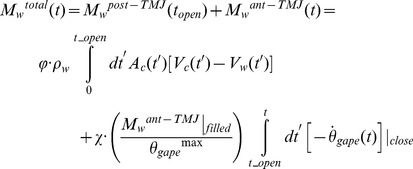
(13b)


#### Equations of motion

The fact that most of the engulfed water is moving in the forward direction motivates the use of the simplest fluid dynamics model possible, namely one-dimensional hydrodynamics. This model is then coupled with the trajectory modeling of the whale’s body as follows [26]:
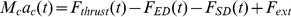
(14)

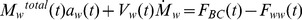
(15)


The accelerations *a_c_* and *a_w_* are those of the whale’s body (*M_c_*) and engulfed mass, respectively. The accelerations are defined from the velocities *V_c_(t)* and *V_w_(t)* measured from a fixed reference frame, *via a_c_ = dV_c/_dt* and *a_w_ = dV_w/_dt*. The filling rate (M-dot) in equation 15 is given by either Equation 12a or 12b. The forces applied on each body all appear on the right-hand-side of both equations; these are further explained in the following sections.

As shown in Figure 3, equations 14 and 15 are used to compute the accelerations *a_c_* and *a_w_* (and then the speeds *V_c_* and *V_w_*). This is done once the gape angle and engulfed mass rates have been calculated via equations 6 and 12a respectively (or equations 7 and 12b during mouth closure), and also once the forces shown in Figure 2 are calculated. The numerical scheme used for this step is discussed in further details by Potvin et al. [26].

These equation of motion also yield a computation of the lead slug position *X_c_(t) – X_w_(t)* (i.e., the very first engulfed slug), which is a useful diagnostic for monitoring the filling state of the cavity post-TMJ. The calculation is performed via the numerical integration of *X_c_(t) – X_w_(t) = ∫dt’ (V_c_(t’) V_w_(t’))*. This observable is used to terminate BLF simulations (with an error message) whenever *X_c_ – X_w_* exceeds the length *L_0_– L_jaw_* of the VGB posterior to the TMJ, an indication that the lead slug is about to accomplish the biologically impossible feat of traveling past the umbilicus.

#### VGB push force (*F_BC_*) and engulfment drag (*F_ED_*)

SE involves the active use of musculature within the VGB and tail in order to set (and keep) the engulfed mass into a state of forward motion. Moreover, such push-forward is metered in a manner to avoid premature cavity filling or premature cavity draining. (The former occurs if the push is too weak and the latter if it is too strong). Such control is possible morphologically given the preponderance of both fast- and slow-twitch muscle within VGB tissue, running from the umbilicus to the snout in bundles layered longitudinally and obliquely [19]. This muscle is thought to resist the lengthening of the VGB posterior to the TMJ so to gradually impart with forward speed the mass being engulfed. Moreover, there are two large “paratendinous” cords emerging out of the mandibular symphysis and project posteriorly in a direction parallel to each mandible [82]. Each cord lies within the buccal cavity walls, just in between the blubber and the ventral most muscle layer. This structure, termed the Y-shaped fibrocartilage skeleton [82], is clearly visible during feeding when the buccal cavity is inflated [84]. It has been hypothesized that this structure adds rigidity to the buccal cavity and acts like a tendon to transmit force from the buccal cavity to the mandibles [82].

The push-forward of the engulfed mass is modulated so to avoid premature cavity filling or draining. Several mathematical forms of the VGB muscle action *F_BC_* have been discussed elsewhere [26]. The most interesting candidate is expressed in terms of the product of an acceleration scale (in brackets) and a mass scale:
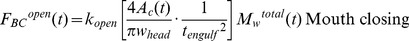
(16)

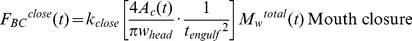
(17)


Cavity wall force gives rise to engulfment drag *F_ED_* (*F_ED_* = *F_BC_*) by action-reaction, and adds to the so-called shape drag *F_SD_* being produced by the flow deflected around the whale’s body (as discussed in the next section). The function *A_c_(t)* is calculated from equations 11 and 6 (or equation 7 during mouth closure), parameter *t_engulf_* from equation 8, and total engulfed mass *M_w_^total^* from equations 13a or 13b.

The reaction constants *k_open_* and *k_close_* (no dimensions) determine how “hard” the engulfed mass is being pushed forward by muscle action: too high of a value and the simulation yields cavity draining as *V_c_(t)* < *V_w_(t)*; too small of a value and the cavity post-TMJ fills up too soon, i.e., prior to maximum gape. In this new version of the BLF model, the values for *k_open_* at each body size (Tables 1, 2, 3, 4) are determined via trial simulations so to obtain *V_c_(t) = V_w_(t) at* the time of maximum gape, thus ending the filling of the cavity post-TMJ at maximum gape *per* the *equivelocity* constraint. Such input *k_open_* are single-valued in that slightly-off values yield either *V_c_(t_open_) < V_w_(t_open_)* and cavity drainage post-TMJ during the entire mouth closure stage; or *V_c_(t_open_) > V_w_(t_open_)* and continued filling of the cavity post-TMJ during closure.

Numerical experimentation suggests values of *k_close_* to be set according to ratios independent of body size, but still varies relating to species specific morphology as *k_open_/k_close_* = 1.49, 1.26, 1.82 and 1.83 for minke, humpback, fin and blue whales respectively (Tables 1, 2, 3, 4). Different values for *k_open_* and *k_close_* make sense morphologically given that the mouth-opening torque applied to the mandibles (mainly by the longitudinal VGB musculature) is maximum during the mouth-opening stage when buccal cavity wall forces are at their largest [26]. It follows that such action must be much weaker during mouth closure in order to minimize that same (mouth-opening) torque that would then be working against the mouth-closing skull musculature [27]. The required forward-push of the engulfed mass could be provided during mouth-closure by VGB oblique musculature which can generate a force without opening the mouth, as done during the filtering/recovery stage (it is noted that such oblique muscle is likely to also assist the longitudinal muscle in providing cavity wall push during the mouth-opening stage). Note that these *k_open_/k_close_* ratios do not guarantee *V_c_ (t) = V_w_(t)* throughout mouth closure; this requirement is enforced instead through a definition of the force *F_ww_* applied on the engulfed mass (Figure 2) as further discussed below. Note also that in previous version of the BLF model [24]–[26] the value of *k_close_* is equal to zero while that of *k_open_* is set to yield *maximum* capacity *M_w_/M_c_* in the cavity post-TMJ (equation 9) by the end of engulfment.

Defining the cavity wall force as a product of an acceleration scale and mass scale leaves a certain degree of arbitrariness in the choice of these scales. Consequently, and although constrained by the *equivelocity* constraint, the values of *k_open_* and *k_close_* listed in Tables 1, 2, 3, 4 are associated with the particular choice of acceleration and mass scales used in equations 16 and 17. In conjunction with this degree of arbitrariness, it is interesting to realize that the alternative definition *F_BC_* = (4 *A_c_(t))/(π w_head_ τ^2^)M_w_(t)* in which *τ* ≡ *t_engulf_*/√ *k_open_* brings up another time scale of interest. Even though the resulting force is obviously identical to that of equations 16 and 17, it suggests another fundamental time scale relevant to engulfment which, as shown in Figure 7, is more insensitive to body size than engulfment time (*t_engulf_*) in addition of being three times shorter (∼ 1 to 2 seconds). Whether this time scale *τ* reflects a property of VGB musculature rather than a constraint on cavity filling (and initial speed *V_c_(0)*) isn’t entirely clear at this time.

#### Shape drag (*F_SD_*)

The drag associated with the flows deflected around an (inflating) body can always be calculated from the general form *F_SD_ = ½ ρ C(t)S(t)V_c_^2^(t)* in which the values of the time-changing drag coefficient *C(t)* are chosen to yield the best match with tag data (Potvin et al. [26]). The time-dependent *drag area C(t)S(t)* represents the combined effects of the accelerating (and decelerating) external flows created around the whale (i.e., the so-called *added* or *apparent* mass), as well as the effects of wake growth and wake turbulence that are generated during the lunge [26]. Although conceptually correct, this approach introduces large uncertainties from the lack of knowledge on the function *C(t)*. Here an alternative but equivalent formulation of shape drag - the so-called *apparent mass* formulation - is used to reduce such uncertainty by including unsteady flow effects that are known to occur, namely those of the deflected fluid masses co-decelerating with the whale:
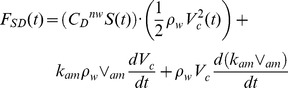
(18)


The drag area *C_D_^nw^S(t)* is defined from a known reference surface area *S(t)* and from a drag coefficient *C_D_^nw^* which is typically *not* equal to the steady-velocity value [85]. This term accounts for the turbulent near-wake found right behind the body. The other two terms in *dV_c_/dt* and in *V_c_* describe the effects of the deflected flows moving about the near wake and also co-decelerating with the whale. Parameter *V_am_(t)* is a reference volume for the fluid accelerated around the body. Such volume may change over time as with the cases of opening parachutes [85]. On the other hand, *k_am_* parameterizes the fraction of added mass that co-accelerates with the body. Since not all the surrounding fluid is doing so at the rate of *dV_c_/dt*, *k_am_* measures (roughly) the equivalent amount of fluid that would be doing so in order to achieve the same drag. With decelerating bodies at high Reynolds numbers, *k_am_* is a complicated function of time which may be strongly dependent on the motion’s history prior to the deceleration [86]–[88]. This contrasts with the low Reynolds number regime in which *k_am_* is a constant and dependent only on the shape of the body [89]. In most cases *k_am_* must be determined empirically and *verified as time-independent* for the system at hand [87]. The added mass formulation has been used previously in comparative bio-mechanics, most notably with the study of shrimp and medusan swimming [90]–[92].

The BLF makes use of the *k_am_* -values associated with ellipsoids, dirigibles and blimps [91], [93], [94], which for the typical rorquals body aspect ratio of 1 to 3 (i.e., aspect ratio defined as buccal cavity length over width (filled)), would yield *k_am_* ∼ 0.2. Here the reference volume of the added mass (*V_am_*) is approximated by a half-ellipsoid of revolution defined by semi-minor axes *L_0_/2* and *½(w_head/_2+ L_jaw_)*.

Note that the added mass formulation embodied by equation 18 is not unique, as both terms in *V_c_* end *V_c_^2^* are derived from a deflection rate of the incoming fluid mass [95]. Depending on the specific accounting of what constitutes the near wake (for the *V_c_^2^* term) and what constitute the co-decelerated flows off-wake (the *k_am_ V_c_* term), different models, and hence different values of *k_am_*, *V_am_ C_D_^nw^* and *S(t)*, can be used to yield the same force. For example, the *k_am_ V_c_* -term can be shown to behave as a *V_c_^2^*–term when the cavity is inflating mostly transversely at rates that are proportional to *V_c_*:
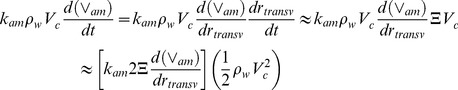
(19)


(*r_transv_* is the mouth radius and Ξ a proportionality constant).

The added mass drag model embodied in the BLF takes the following forms:

- *Mouth opening*.
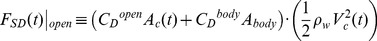
(20)


- *Mouth closure*.
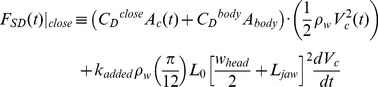
(21)


The *k_am_ dV_c_/dt* term is neglected in equation 20 given the low body (whale) accelerations and engulfed volumes at play during mouth opening [J. Potvin; unpublished data]. In contrast, this term is quantitatively important during mouth closure and, with *dV_c_/dt* being negative (a deceleration), approximately reflects the effects of the near wake re-contact the body by actually *reducing* shape drag. Parameter *A_body_* is the known maximum cross-sectional area of the body in a closed-mouth, empty-cavity configuration (Figure 1), *A_c_(t)* the instantaneous mouth area (equation 11) and *A_c_^max^* the maximum mouth area (equation 11 with *θ_gape_^gape^* = 50° or 78° ). *C_Dbody_* corresponds to the drag coefficient for a mouth-closed and empty-body configuration, set to *C_Dbody_* ^= ^0.05 from a previous hydrodynamic study on fin whale locomotion [37]. Although the drag coefficients *C_D_^open^* and *C_D_^close^* were assigned the values shown in Tables 1, 2, 3, 4 (∼ 0.3 to 0.5), the resulting accelerations and speeds turned out to be insensitive to their specific values, in contrast to the simulations discussed in [24], [25]. This is particularly true during mouth closure when shape drag is exactly cancelled by fluking thrust as discussed next.

#### Swimming thrust (*F_thrust_*), buoyancy-reduced weight (*F_ext_*), and “ocean-to-engulfed mass” drag force (*F_ww_*)

The remaining forces acting on the body and engulfed mass are as follows (Figure 2): swimming thrust (*F_thrust_*); buoyancy-reduced weight (*F_ext_*; component along the track); and the “ocean-to-engulfed mass” drag force (*F_ww_*), which acts on the ocean-facing end of the engulfed mass. Calculating their values during engulfment can only be estimated as they have never been experimentally characterized. The buoyancy-reduced weight is defined as *F_ext_ = (F_B_ – W_c_) sinΘ*, with *F­_B_*, *W_c_* and *Θ* corresponding to a whale’s buoyancy, weight and angle of the upward-inclined trajectory respectively (Note: at depth *F­_B_* < *W_c_*, a case of negative buoyancy). On the other hand, *F_ww_* arises from the pressure applied by the oncoming flow under the palate to the ocean-facing end of the engulfed water (Figure 2). In a previous version of the BLF both *F_thrust_* and *F_ext_* were estimated from tag data as being quite small relative to engulfment drag (*F_ED_*) and set to zero values (see Appendix 1 in Goldbogen et al [24]); similarly, *F_ww_* was set to zero. In this paper we use non-zero values which are still small but yield better matches with the (speed) tag data of the three species, in addition to yielding a more realistic picture of the overall fluid motion.

### Mouth Opening Stage

The combination *F_thrust_* + *F_ext_* is modeled simply by using a constant value throughout that stage, presumably the average of the actual (but unknown) time-varying force. For the size corresponding to the average body length of humpback whales (14.4 m), fin whales (20.2 m), and blue whales (25.2 m), *F_thrust_* + *F_ext_* is set to a value that yields the best match with the speed data of tag studies [26], [27]. Such values (Tables 1, 2, 3, 4) are then scaled all other body dimensions as follows:

- Blue whale.
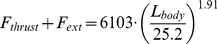
(22)


- Fin whale.
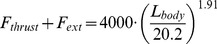
(23)


- Humpback whale.
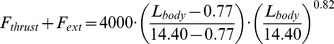
(24)


These scaling laws are based on assuming *F_thrust_* being much greater than *F_ext_*, and by using a simple model in which *F_thrust_* is given by fluke area times flow speed relative to the fluke -squared. As discussed in more details in the very last section of this paper, *F_thrust_* scales mostly with fluke area since the flow speed relative to it is driven mostly by the tangential speed of the fluke along the arc of tail sweep, a variable which turns out to scale weakly with *L_body_*. Note that the *F_thrust_*-values obtained from equation 21 are generally lower than those obtained from the Bose-Lien model used in the RAAMR calculation (at the same speed and body size). This should follow from the intermittent short bouts of fluking that occurs during mouth opening. On the other hand, the speed of the reflux being generally low during most of the mouth opening stage means that little pressure is felt by the ocean-facing end of the engulfed mass (residing just below the TMJ). Thus the ocean-to-engulfed mass drag force is set to.
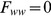
(25)


### Mouth Closure Stage

The *equivelocity* condition required by SE, as applied to the water in both cavity sections, makes the interaction between whale and engulfed water look like a perfectly inelastic collision. In elementary college physics, such a collision is exemplified by two balls moving towards each other in the absence of external forces, then colliding and moving together while permanently attached. Here, and in the presence of external forces, a perfectly inelastic collision is implemented to yield *V_c_ = V_w_* and *a_c_ = a_w_*, or in other words, *equivelocity* and *equideceleration*, by solving equations 14 and 15 under the following constraints:
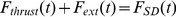
(26)

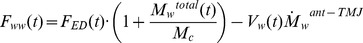
(27)


In this scheme the forces *(F_thrust_* +*F_ext_)* and *F_ww_* are calculated from the knowledge of the mass so-far engulfed (equation 13), the reflux speed (equation 15), the filling rate (equation 13), and both shape and engulfment drag (equations 16–17 and 20–21).

Although these constraints are not unique in yielding equideceleration, they represent a plausible scenario in which the whale applies swimming thrust in amounts that are just enough to keep up with the deceleration of its engulfed cargo, which itself is caused by the push differential between buccal cavity force (*F_BC_*) and the back-end pressure from the ocean (*F_ww_*). In this case thrust is modulated to counteract the decelerating effects of shape drag and negative buoyancy. Interestingly, the body size scaling of swimming thrust now reflects that of shape drag as with the case of steady travel. Moreover, even though any shape drag model becomes irrelevant to the actual motion of the whale (being cancelled by *F_thrust_* + *F_ext_*), they remain quite relevant to metabolic expenditures as they become a driver of swimming thrust (during mouth closure).

#### More results; scaling of engulfment time and peak cavity wall force

**Figure 13:**
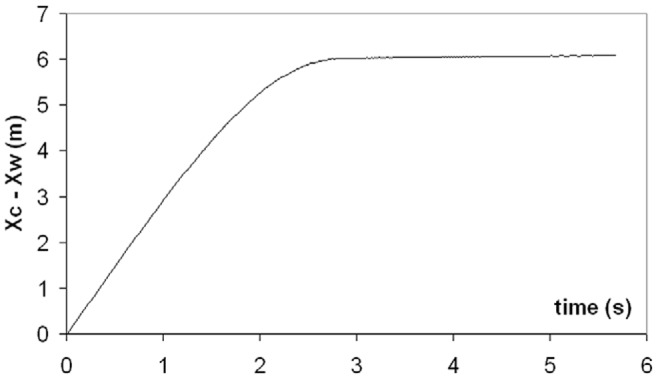
Lead slug location with respect to the TMJ. Simulated positions of the first-engulfed slug for a 20.2 m fin whale. Here the lead slug stops traveling beyond the time of maximum gape (i.e. after the 2.85 s mark), *per* Synchronized Engulfment. Note also that this slug can never travel past the umbilicus, or in other words, cover a distance exceeding *L_0_– L_jaw_* ( = 7.34 m in this case).

**Figure 14:**
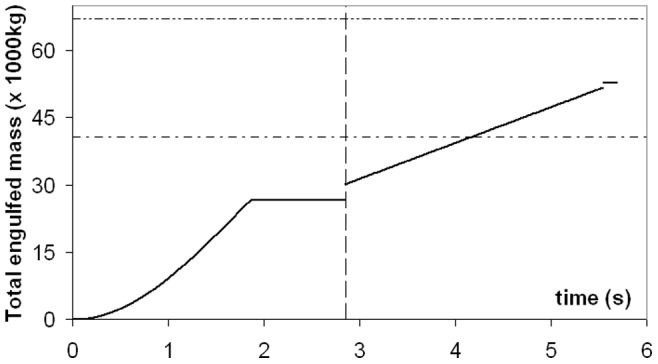
Simulated temporal variation of the engulfed mass for a 20.2 m fin whale. The cavity post-TMJ fills first during the first 66% of the mouth-opening (i.e., until 1.9 s here), to remain filled thereafter. This is followed by the filling of the cavity ant-TMJ during mouth closure. These are compared with the total mass capacity of the cavity post-TMJ (equation 9; dash-dotted line) and total capacity (sum of equations 9 and 10; dash-doubly-dotted). The body mass of this case is 40,705 kg.

Typical BLF results are shown in Figures 5, 6, 7 previously discussed. More are displayed in Figures 13 and 14, which show the temporal changes in the position of the lead slug of water (or first-engulfed slug) and of the mass so-far engulfed. In Figure 13 the lead slug’s motion clearly ends prior to maximum gape (*t_engulf_/2*) in accordance with SE. Such motion stops a meter or so away from the aft-end of the cavity located at a distance *L_0_– L_jaw_* posterior to the TMJ, a feature designed for avoiding cavity drainage during the rest of engulfment. The extent of the partial filling post-TMJ is also shown in Figure 14. Note that the modeling allows the cavity ant-TMJ to fill completely in contrast to the cavity post-TMJ.z

**Figure 15:**
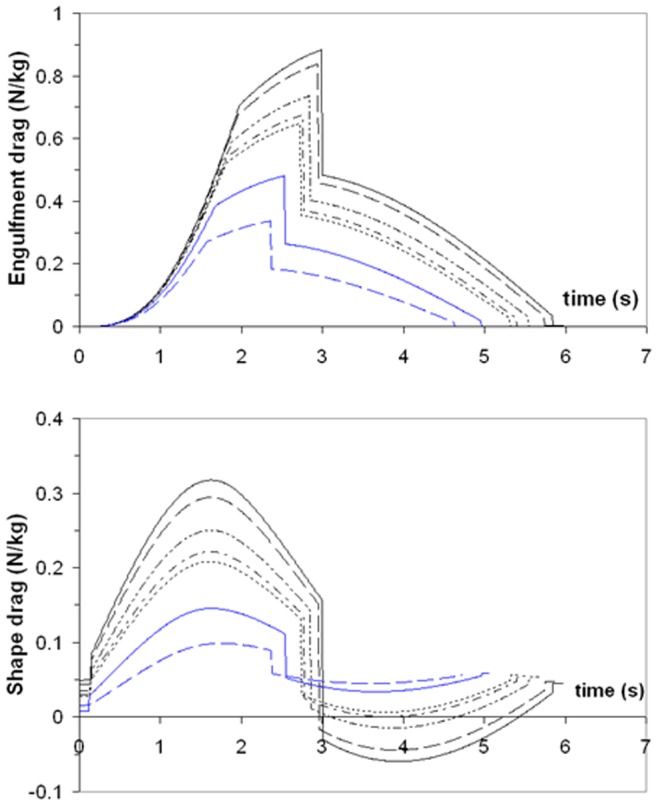
Simulated engulfment (top) and shape drag (bottom) by fin whales (mass-specific). Temporal development at various body lengths: 24.0 m (black, continuous), 22.7 m (black, dashed), 20.2 m (black, dashed-doubly-dotted), 18.5 m (black, dashed-dotted), 17.7 m (black, dotted), 13.5 m (blue, continuous), 10.0 m (blue, dashed).

The simulations show how engulfment forces and times change with body size (Figures 6, 7 and 15, and Tables 5, 7 and 8), as well as how they are constrained by: 1) the scaling of *Synchronized Engulfment*, 2) the allometry of the engulfment apparatus [23], and 3) the initial lunge speed (*V_c_(0) = V_n_ L_body_* [24]). Interestingly, these three elements contribute in different ways for each engulfment performance measures.

Engulfment time reflects the opening and closing (angular) speeds of the mouth as driven by the escape speeds of the prey [27]. Elementary dimensional analysis suggests engulfment time to scale as a ratio of length over speed [68], [97], or in the context of engulfment, as *t_engulf_* ∼ *L_jaw_/V_c_(0)*. A recent analysis of tag data for all three species of large rorquals [24], as well as plausible prey escape scenarios [27], suggest the initial velocity to scale as *V_c_(0) ∼ 0.15 L_body_* which in turns implies *t_engulf_* ∼ *L_jaw_/L_body_*. Using the morphological data of Tables 1, 2, 3, 4 where *L_jaw_ ∼ L_body_^1.21, 1.29 and 1.41^* for humpback, fin and blue whales, respectively, engulfment time would ultimately scale as *t_engulf_* ∼ *L_body_^0.21, 0.29 and 0.41^*. Interestingly, this is quite similar to the scaling of SE-constrained engulfment time of equation 8 for which *t_engulf_* ∼ *L_body_^0.20, 0.27 and 0.41^* (Table 2; see also Figure 7). This comparison shows that even though SE contributes a non-trivial dynamical scaling factor to engulfment time (namely, the factor *Γ(L_0_– L_jaw_)/L_jaw_* in equation 8), the basic allometry of the skull in which *L_0_ ∼ L_body_^1.18, 1.16 and 1.19^* (Tables 1, 2, 3, 4) significantly weakens its significance to levels usually expected with isometric body dimensions. This is an important “non-contribution” given the body size range examined here, from weaned juvenile humpback whales (8 m) up to the largest adult blue whales (27 m).

Mass-specific peak cavity push (or peak engulfment drag) at maximum gape is a measure for which SE is somewhat more important. As shown in Figure 6, and per Equation 11, the increase in mass-specific cavity wall force is mostly the result of the positive allometry of the skull which here directly increases mandibular size (*L_jaw_*) as well as engulfment capacity (*M_w_^post-TMJ^*). With *t_engulf_* scaling as previously discussed, and with *M_w_^post-TMJ^*, *M_c_* and *A_c_/w_head_* scaling as *L_body_^3.43, 3.66 and 3.80^* (equation 9), *L_body_^3.00, 2.74 and 3.46^* (Tables 1, 2, 3, 4) and *L_body_^1.21, 1.29 and 1^.* ^41^ (i.e., as ∼*L_jaw_*; see equation 11) respectively, the product (*A_c_/w_head_*)(*M_w_^post-TMJ^*/*M_c_*) thus varies as *L_body_^1.23, 1.67 and 0.93^* and so drives the scaling of the ratio as *F_BC_^open^|_peak_*/*M_c_* as ∼ *L_body_^1.14, 1.09 and 0.75^* (Table 5). The difference resides in the scaling of the reaction constant *k_open_* appearing in equation 16, which is tuned at each body size to enforce *equivelocity* at maximum gape. The relatively weaker scaling of *k_open_* (∼ *L_body_* ^−*0.36,*^ ^−*0.61,*^ ^−*0.35*^; Tables 1, 2, 3, 4) shows again the reduced role of SE in comparison to morphology. This is also seen with the mass-specific expended power, where the SE-dependent effects connected to the scaling of *k_open_* are neutralized by those of the (average) speed (also SE-dependent).

#### Scaling of fluking thrust during engulfment

The scaling of the fluke thrust (*F_thrust_*) embodied in equations 20 and 21, although seemingly simple, is by no means straightforward. It is usually derived in the context of non-feeding steady travel (at speed *V_cruise_*) during which body drag is equal to fluking thrust (in this context “body drag” is synonymous with “shape drag”). Thus *F_thrust_ ∼ L_body_^2^ V_cruise_^2^* with an assumption of the drag coefficient being weakly dependent on body size [70]. In cases where the cruise speed is determined by minimal energy expenditure via the use of a resonance related to tail stiffness, one has *V_cruise_ ∼ L_body_^1/2^* and *F_thrust_ ∼ L_body_^3^* [96]. On the other hand, where there is a need for the differently-sized members of a group travelling together to move at the same speed, fluking thrust is applied differently by each member so to yield *V_cruise_ ∼ L_body_^0^* and *F_thrust_ ∼ L_body_^2^*. Using body drag as basis for studying the scaling of fluking thrust no longer applies for engulfment given that the associated motions are fundamentally unsteady, or in other words, given that thrust is equal to body drag *in addition to* the inertial force (i.e, -*M_c_a_c_*) and engulfment drag (*F_ED_*).

**Figure 16:**
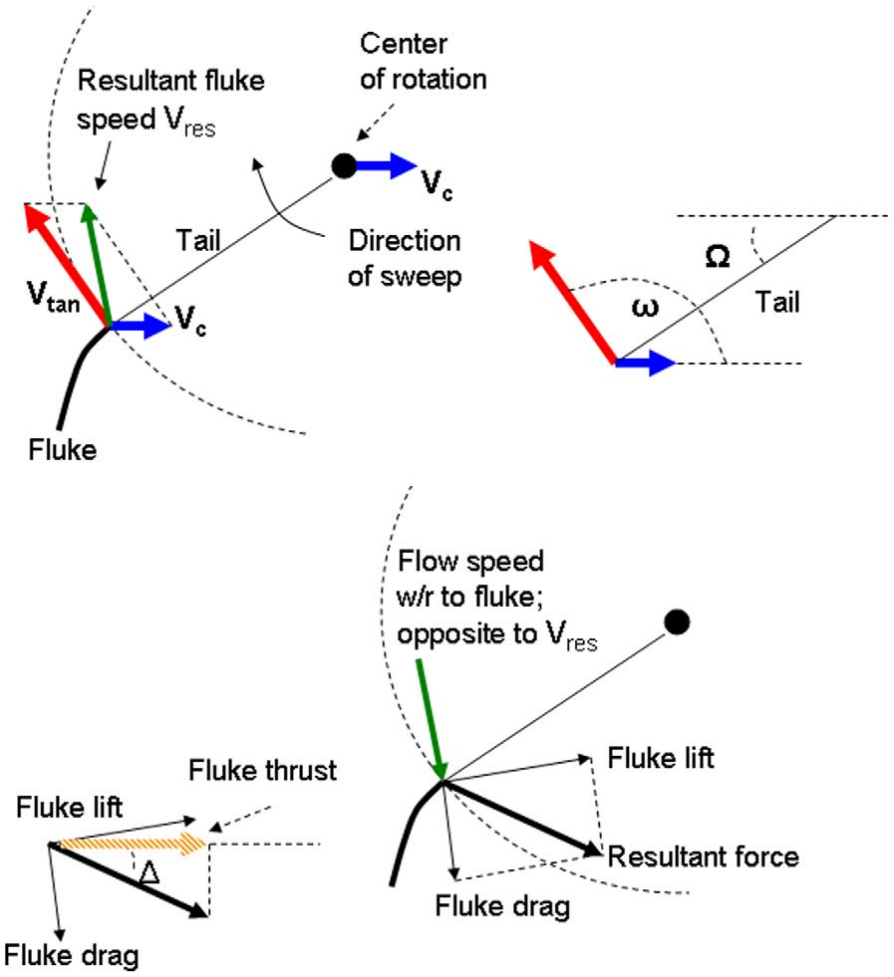
Simple model of the forces and velocities about the flukes.

A much simpler – and fundamental - way to look at thrust is to view the flukes as (flexible) hydrofoils that produce lift and drag forces combining into thrust in the forward direction (Figure 16):
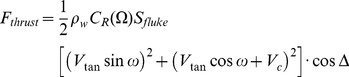
(28)


Here the angles *Ω* and *ω* measure the directions of the tail and fluke’s tangential speed (*V_tan_*) respectively. *C_R_* is the force coefficient resulting from the (vector) addition of both airfoil drag and lift, and Δ the angle made by the resultant with respect to the horizontal. Finally, *S_fluke_* is the *referenc*e area defined as *fluke span* times *fluke chord*.

The flukes’ tangential speed along the arc of a sweep is estimated as *V_tan_ ∼.* *L_tail_ Ω_sweep_*/*t_sweep_*, with *L_tail_ ∼ L_body_/2* and *Ω_sweep_* = 1.57rads (i.e., 90°). The sweep duration *t_sweep_* is informed by the trends seen in tag data collected on the three species [24], [25], [34], [35], which suggests that one fluking sweep early in the mouth opening stage (either upwards or downwards) lasts about 1/3 of engulfment time (or *t_sweep_ ∼ 1/3 t_engulf_* ). In the case of the average body sizes shown in Tables 1, 2, 3, 4, one has *t_engulf_ = *4.1s (humpback), 5.69 s (fin) and 6.42s (blue), yielding *t_sweep_ = *1.36s (humpback), 1.89s (fin) and 2.33s (blue). With *L_tail_* being equal to 7 m (humpback), 10 m (fin) and 13 m (blue), one has *V_tan_ ∼* 8.1 m/s (humpback), 8.3 m/s (fin) and 8.76 m/s (blue). This tangential velocity turns out to be greater than the whale’s translation speed (*V_c_*) thus leading to *V_tan_^2^>> V_c_^2^* and to the neglect of the *V_c_^2^*–term in equation 26. This result means that the velocity-squared factor of the equation is roughly independent of body size. After again assuming the aerodynamic force coefficient *C_R_* as independent of body size, fluke thrust thus scales as *F_thrust_ ∼ S_fluke_ V_tan_^2^*.

Understanding why *V_tan_* scales weakly with body size can be achieved by realizing that *V_tan_* ∼ *L_body_/t_sweep_ ∼ L_body_/L_body_ ∼ 1*. The last step follows from an argument similar to Hill’s estimate of muscle contraction frequency under maximum stress, as discussed by Pennycuick [97]. Thus *F_thrust_ ∼ S_fluke_*, and although *F_thrust_ ∼ L_body_^2^* in an isometric world, fluking thrust scales somewhat more weakly in the real world of rorquals [23]. Indeed, scaling the fluke reference area in terms of the fluke span (*K_span_*) and fluke width at insertion (i.e., *K_WAI_*, measured from notch of fluke to medial insertion [23]) one obtains for both fin and blue whales *S_fluke_ ∼ K_span_K_WAI_* ∼ *L_body_^1.07^ L_body_^0.84^* [23], and *S_fluke_ ∼ K_span_K_WAI_ ∼ (L_body_ –0.77) L_body_^0.82^* for humpback whales, which then lead to the scaling laws of Eqs. 20–22 (for *K_span_* see [98]; scaling for *K_WAI_* is from J. Goldbogen (unpub. data)).

## Supporting Information

Text S1 A glossary of symbols and acronyms.
(DOC)
