# Supplementary material for: Metabolic Expenditures of Lunge Feeding Rorquals Across Scale: Implications for the Evolution of Filter Feeding and the Limits to Maximum Body Size
Source: PLoS One. 2012 Sep 14;7(9):e44854. doi: 10.1371/journal.pone.0044854 (PMC3443106; doi:10.1371/journal.pone.0044854)
Supplement: Text S1 — A glossary of symbols and acronyms. (DOC) [file pone.0044854.s001.doc]

**Supporting Information**

**List of Symbols and Acronyms**

**Symbols**

*a_c_(t) =* Whale acceleration or deceleration

*a_w_(t) =* Engulfed mass acceleration or deceleration

*A_body_ =* Cross-section area (at TMJ axial location) of empty whale

*A_c_(t) =* Cross-section area (at TMJ axial location) of buccal cavity during engulfment

*C(t)S(t) =* Generic (shape) drag area

*C_D_^body^ =* Shape drag coefficient of the body during non-feeding travel

*C_D_^close^ =* Shape drag coefficient of the body during mouth-closing

*C_D_^nw^ =* Unsteady drag coefficient used in equation 18

*C_D_^open^ =* Shape drag coefficient of the body during mouth-opening

*C_R_ =* Force coefficient of the combined lift and drag forces generated by the flukes

*dt =* Simulation time increment

*F_ext_ =* Generic external force applied to a whale (including weight and buoyancy)

*F_D_^steady^ =* Drag generated by the whale during non-feeding (steady) travel

*F_BC_ =* Force applied on the engulfed mass by the buccal cavity walls

*F_ED_ =* Engulfment drag

*f_maxlunge_=* Maximum number of lunges in a dive

*F_SD_ =* Shape drag

*F_thrust_=* Fluke thrust

*F_thrust_^steady^ =* Steady-state fluke thrust obtained by Bose and Lien [26] for a 14.5m fin whale

*F_ww_=* “Ocean-to-engulfed mass” drag

*h_sync_ =* Scaling factor in the engulfment duration equation (equation 8)

*k_am_ =* Apparent (or “added”) mass coefficient

*k_close_ =* Reaction constant (mouth-closing stage)

*k_open_ =* Reaction constant (mouth-closing stage)

*L_0_ =* Length of the VGB

*L_body_ =* Length of the body

*L_jaw_ =* Lateral projected length of the mandibles

*M_c_ =* Flesh mass of the whale (i.e., mass of the whale *pre-engulfment*)

*M_w_(t) =* Mass of the engulfed water (instant value)

*M_w_^post-TMJ^* and *M_w_^ant-TMJ^ =* Maximum capacities of the cavities posterior and anterior to the TMJ

*P_T_^metab^*= Metabolic power expended for performing the mechanical work by *F_thrust_*

*P_VGB_^metab^*= Metabolic power expended for performing the mechanical work by *F_ED_*

*P_transit_^metab^*= Metabolic power expended for performing the mechanical work by *F_thrust_^steady^*

*r_trans_(t) =* Instant cavity radius used in equation 19

*S_fluke_ =* Fluke surface area

*t =* Time

*t_ascent_ =* Ascent time from foraging depth during a dive

*t_close_ =* Duration of the mouth-closure sub-stage

*t_descent_ =* Descent time from foraging depth during a dive

*t_engulf_ =* Duration of engulfment (i.e., mouth opening & closure combined)

*t_filter_ =* Duration of an entire lunge

*t_lunge_* = Duration of a single lunge (= *t_pa_* + *t_engulf_* + *t_filter_*)

*t_open_ =* Duration of the mouth-opening sub-stage

*t_maxdive_ =* Maximum diving time

*t_pa_ =* Duration of the prey-approach stage

*t_search_ =* Search time for prey in between lunges (during a dive)

*w_head_ =* Width of the head

*V_c_(t) =* Speed of the whale with respect to a fixed reference

*V_flow_ =* Average speed of the 3-dimensional flow within the cavity

*V_n_* = Specific initial speed (= *V_c_(0)/L­_body_*)

*V_w_(t) =*Speed of the water engulfed within the cavity with respect to a fixed reference

*V_tan_ =* Fluid speed tangential to the flukes

V_am_ = Added mass reference volume

*X =* Mass fraction of body parts expending energy at a rate equal to AMR

*X_jd_ =* Jaw disarticulation factor

*X_c_ =* Location of the whale (TMJ) with respect to a fixed reference

*X_w_ =* Location of the lead engulfed slug with respect to a fixed reference

*Y* = Mass fraction of body parts expending energy at a rate equal to BMR

**Greek symbols**

*Δ =* Angle of the flukes’ lift-drag resultant with respect to the horizontal (Figure 16)

*ΔE_O2_* = Energetic contents in the blood stored in muscle, blood and lungs

*ΔQ_T_^mech^*= Mechanical energy expended by *F_thrust_*

*ΔQ_VGB_^mech^*= Mechanical energy expended by *F_ED_*

*ΔQ_T_^metab^*= Metabolic energy expended by *F_thrust_*

*ΔQ_VGB_^metab^*= Metabolic energy expended by *F_ED_*

*ΔX_c_ =* Distance travelled by the whale during time interval *V_c_ dt*

*χ =* Filling efficiency (buccal cavity ant-TMJ)

*Ξ* = Proportionality constant in equation 19

*φ =* Filling efficiency (buccal cavity post-TMJ)

*Γ =* Scaling factor in the engulfment duration equation (equation 8)

*ω* = Angle between *V_tan_* and *V_c_* (in Figure 16)

*Ω* = Tail angle with respect to the horizontal

*ρ_w_ =* Salt water density

*θ_gape_ =* Gape angle

*θ_gape_^max^ =* Maximum gape angle

*τ =* Time scale of the *shove* (equation 16)

**Acronyms**

AMR = Active metabolic rate

Ant-TMJ = Anterior to the temporomandibular joint

BLF = Basic Lunge Feeding (model)

BMR = Basal metabolic rate of marine mammals

BMR(terr) = Basal metabolic rate of terrestrial mammals

EMR = Engulfment metabolic rate

EMR* = “Instant” engulfment metabolic rate, i.e. calculated over 0.1s intervals

MMR = Maximum metabolic rate

PAMR = Prey-Approach Metabolic Rate

Post-TMJ = Posterior to the temporomandibular joint

RAAMR = Rorqual average active metabolic rate

F/RMR = Metabolic rate during the filter and recovery stage

SE = Synchronized engulfment

TADL = Theoretical aerobic diving limit

TMJ = Temporomandibular joint

VGB = Ventral groove blubber
